# Exploring vulnerability to heat and cold across urban and rural populations in Switzerland

**DOI:** 10.1088/2752-5309/acab78

**Published:** 2023-02-14

**Authors:** Evan de Schrijver, Dominic Royé, Antonio Gasparrini, Oscar H. Franco, Ana M. Vicedo-Cabrera

**Affiliations:** 1 Institute of Social and Preventive Medicine (ISPM), University of Bern, Bern, Switzerland; 2 Oeschger Center for Climate Change Research (OCCR), University of Bern, Bern, Switzerland; 3 Graduate school of Health Sciences (GHS), University of Bern, Bern, Switzerland; 4 Department of Geography, University of Santiago de Compostela, Santiago de Compostela, Spain; 5 CIBER of Epidemiology and Public Health (CIBERESP), Spain; 6 Department of Public Health, Environments and Society, London School of Hygiene & Tropical Medicine, London United Kingdom; 7 Centre on Climate Change and Planetary Health, London School of Hygiene & Tropical Medicine, London (LSHTM), London, United Kingdom; 8 Centre for Statistical Methodology, London School of Hygiene & Tropical Medicine, London United Kingdom

## Abstract

Heat- and cold-related mortality risks are highly variable across different geographies, suggesting a differential distribution of vulnerability factors between and within countries, which could partly be driven by urban-to-rural disparities. Identifying these drivers of risk is crucial to characterize local vulnerability and design tailored public health interventions to improve adaptation of populations to climate change. We aimed to assess how heat- and cold-mortality risks change across urban, peri-urban and rural areas in Switzerland and to identify and compare the factors associated with increased vulnerability within and between different area typologies.

We estimated the heat- and cold-related mortality association using the case time-series design and distributed lag non-linear models over daily mean temperature and all-cause mortality series between 1990-2017 in each municipality in Switzerland. Then, through multivariate meta-regression, we derived pooled heat and cold-mortality associations by typology (i.e. urban/rural/peri-urban) and assessed potential vulnerability factors among a wealth of demographic, socioeconomic, topographic, climatic, land use and other environmental data.

Urban clusters reported larger pooled heat-related mortality risk (at 99th percentile, vs. temperature of minimum mortality (MMT)) (relative risk=1.17(95%CI:1.10;1.24, vs peri-urban 1.03(1.00;1.06), and rural 1.03 (0.99;1.08)), but similar cold-mortality risk (at 1st percentile, vs. MMT) (1.35(1.28;1.43), vs rural 1.28(1.14;1.44) and peri-urban 1.39 (1.27-1.53)) clusters. We found different sets of vulnerability factors explaining the differential risk patterns across typologies. In urban clusters, mainly environmental factors (i.e. PM_2.5_) drove differences in heat-mortality association, while for peri-urban/rural clusters socio-economic variables were also important. For cold, socio-economic variables drove changes in vulnerability across all typologies, while environmental factors and ageing were other important drivers of larger vulnerability in peri-urban/rural clusters, with heterogeneity in the direction of the association.

Our findings suggest that urban populations in Switzerland may be more vulnerable to heat, compared to rural locations, and different sets of vulnerability factors may drive these associations in each typology. Thus, future public health adaptation strategies should consider local and more tailored interventions rather than a one-size fits all approach. size fits all approach.

## 1 Introduction

There is a well-established relationship between exposure to non-optimal temperatures and a wide range of adverse health outcomes ^1,2^. Currently, non-optimal temperatures are associated with approximately 9.4% of the total mortality burden globally, which corresponds to 74 deaths per 100,000 people, of which the largest part can be attributed to cold (8.5% versus 0.9% for heat)^2^. Evidence suggests that climate change is already substantially affecting populations leading to an additional heat-mortality burden which is likely to increase further in the future and even overtake the current cold-related mortality under various climate change scenarios ^3,4^. Even with full implementation of the Paris agreement and reaching net-zero carbon emissions by 2050, the inherent inertia of the climate system will continue to increase temperatures for several more decades after^5,6^, yielding a substantial additional health burden ^7,8^. Thus, accelerated adaptation to non-optimal temperature is essential to reduce the heat-related mortality burden ^9,10^. Moreover, besides the expected increase in heat-related mortality, cold-related mortality is likely to further increase due to population ageing, showing the necessity to identify further adaptation strategies and vulnerability factors ^11,12^

A large body of literature has shown that the temperature-mortality association can substantially vary across different geographical units and population sub-groups^13–22^, which are driven by small area characteristics such as access to air conditioning (AC), ageing, greenness and socioeconomic level amongst others^19–21,23–25^. However, most of the existing evidence of temperature-mortality risks and corresponding vulnerability factors rely on impact assessments conducted in urban locations alone, since smaller cities and rural locations have barely been assessed due to a lack of valid exposure data ^26,27^, low statistical power ^28,29^, or have been considered as part of larger regions lacking the local dimension of the risks.

Even though urban areas tend to be warmer than rural regions due to the Urban Heat Island effect (UHI), rural regions have found to be at least equally vulnerable to temperature and climate change^14,17,18,22,30–33^. Moreover, the association between urbanicity and heat-vulnerability has been hypothesised to follow a U-shape curve, with larger risks in extremely urban or extremely rural regions^17^, while for cold, rural regions tend to be more vulnerable due to lower access to health care, lower baseline health or poverty amongst others ^12,16^. Although there is agreement on spatial variability of the temperature-mortality association, little is known regarding differential drivers of temperature-vulnerability between typologies. Moreover, in Switzerland, previous studies have observed large variation in the heat- and cold-related mortality impacts between cities and cantons with larger heat-mortality impacts in urban regions (i.e., Zurich, Basel and Geneva), and larger cold impacts in rural regions. However, thus far the underlying mechanisms for this large spatial variation has remained unexplored and it is not known which social (i.e., climate injustice), biological (i.e., ageing) or environmental vulnerability factors (i.e., particulate matter concentrations and temperature) explain the variation of non-optimal temperature-mortality impacts between regions in Switzerland. Understanding the mechanisms and factors driving vulnerability in urban/rural locations can help to identify the most vulnerable populations and aid the design of tailored public health interventions to modulate heat and cold-related vulnerability.

In this assessment, we hypothesise that vulnerability to heat and cold vary across urban and rural locations driven by different sets of vulnerability factors. First, vulnerability to non-optimal temperature is usually dependent on small-area level characteristics of the population and environment, which are highly heterogeneous within and between urban and rural regions. Second, these characteristics or factors can have different effects in each type of area (i.e., level of greenness in urban vs. rural locations). Therefore, we aimed to assess how heat- and cold-mortality risks differ across urban, peri-urban and rural regions between 1990 and 2017 in Switzerland, and to explore what factors are associated with increased vulnerability to non-optimal temperatures in each type of region. The novelty of this assessment is the application of a recently developed statistical framework to study the effect modification of individual variables in a complex multivariable regression model ^16,34^, using a wealth of sociodemographic and environmental data available at high resolution.

## 2 Methods

### 2.1 Study setting

Switzerland is a country with a particularly sparse population which is unevenly distributed throughout the country (Fig S1). In particular, North and West Switzerland are more highly populated (where the main cities such as Zurich, Basel and Geneva are located) compared to Central and East Switzerland where the Swiss Alps are (Fig S2), creating stark differences in climate, orography and population distribution. Additionally, characteristics and composition of the populations in terms of social, demographic, and environmental factors are widely heterogeneously across Switzerland, with strong differences between urban, peri-urban and rural (Table 1, Fig S3)).

### 2.2 Temperature and mortality data

We collected daily time series data on all-cause mortality and temperature for all 2,054 municipalities in Switzerland between the 1st of January 1990 and the 31st of December 2017. The individual mortality data was provided by the Swiss Federal Bureau of Statics (BFS). We obtained the daily mean temperature on a 1.6 x 2.3 km grid across the full Swiss geographic extent from a gridded climate dataset (MeteoSwiss-grid-product) developed by Meteoswiss. We then derived the corresponding population-weighted average temperature series for each municipality, as described in a previous study^35^. The use of such high-resolution temperature grid cells has shown to be a valid alternative to monitor stations to assess temperature-mortality impacts. It also has many advantages as opposed to monitor stations, as it allows us to assign an exposure to remote areas regardless the presence or not of weather stations (i.e., rural districts).^35,36^

### 2.3 Vulnerability factors

We initially compiled an integrated dataset of 42 variables characterizing the population and environment for each municipality which we believed could modulate the vulnerability to non-optimal temperatures. We included several socioeconomic variables (social index (SES), percentage of new houses, ageing index), topographic variables (access to health care, population density), climatic variables (annual mean temperature and temperature range) as well as land use and environmental data (impervious surfaces, constructed area, Enhanced Vegetation Index (EVI), PM_2.5_ annual concentration, percentage of water area). The full list of variables with the corresponding definition and source is provided in Table S1. These variables were derived for each municipality and then aggregated to a new higher agglomerative cluster level (defined in Section 2.4). The spatial distribution for each variable at municipality level resolution is illustrated in Figure S1 with the correlation between all variables at the district level in Figure S4.

Since many of the 42 variables showed a large degree of multi-collinearity (Figure S3), we reduced dimensionality in two ways. For the assessment of individual effect modification by vulnerability variable (Section 2.6), we selected 9 variables that we considered representative of different features based on the coordinates of the principal component and correlation matrix(Figure S5) (using the correlation matrix between variables by urban/peri-urban/rural clusters is illustrated in Figure S6). The 9 selected variables were used as single vulnerability variables in our assessment as elaborated upon in section 2.6. Then, we conducted a principal component analysis (PCA) over the 9 selected vulnerability factors and created two principal components (PC), explaining the heterogeneity between urban, peri-urban and rural districts, respectively. These two components were then used to account for within-area typology-specific confounders when predicting the pooled urban, peri-urban and rural temperature-mortality association (as discussed in section 2.5).

### 2.4 Definition of urban, peri-urban and rural clusters

We defined a set of urban, peri-urban and rural clusters by combining all of the 2,054 municipalities into 94 higher agglomerative clusters using the Ward-like hierarchical clustering method with geographical constraints using municipality-level information on several vulnerability variables^37^. The Ward-like algorithm is a constrained hierarchical clustering method that aims to optimize a convex combination using two dissimilarity matrices and a mixing parameter to create a new higher agglomerative layer consisting of municipalities that are both similar and proximal to each other (Figure S6)^37^. Methods S1 provides a more elaborate explanation. Subsequently, we developed a new agglomerative level consisting of 94 clusters, which was based on municipalities that were both similar- and proximal to each other and had a minimum of 1,000 deaths. We then classified each high agglomerative cluster as “urban”, “peri-urban” or “rural” according to the following criteria: each municipality was defined as urban or rural based on the official definition of BFS. When <50% of the population in each cluster lived in urban municipalities, we considered the cluster rural, when 50-80% resided in an urban municipality it was considered peri-urban and when >80% of the population resided in an urban municipality, we considered the cluster urban. We consider this ad hoc definition of clusters more appropriate for this study purposes, compared to the administrative upper level (i.e. district) defined by BFS, and also more appropriate than using an ad hoc definition based on clustering variables as used for the Ward-like hierarchical clustering method (i.e. EVI, where highly urbanised regions can have a similar value as mountainous regions). In particular, we believe that the differential effects of vulnerability factors by typology on the temperature-mortality association could be diluted as the Swiss orography, population characteristics (demography, environment) and distribution are highly heterogeneous within districts. Using the proposed approach, the municipalities included in the derived high agglomeration clusters are more homogeneous, thus allowing for a better characterisation of the vulnerability of the population and it would help to better capture the signal of potential effect modification of vulnerability factors.

### 2.5 Estimation of the temperature-mortality associations

To estimate the temperature-mortality association in each cluster, we performed a case time series analysis with conditional quasi-Poisson regression and distributed lag nonlinear models using municipality-specific temperature-mortality series^15,38^. The case time series design allowed us to estimate the exposure-response function within a cluster, but still use the high-resolution municipality level data, therefore, reducing exposure misclassification and increasing the precision of the estimates. This design also controls for temporal trends using a matching stratum defined by year, month and day of the week by municipality. We modelled the cluster-specific temperature-mortality association using the distributed lag non-linear framework, a flexible technique to model non-linear exposure-response associations and lagged dependencies ^39^. We defined a quadratic B-spline with three internal knots placed at the 10^th^, 75^th^ and 90^th^ percentile of the cluster-specific temperature distribution (Table S2). We modelled the lag-response curve using a natural spline with three internal knots equally placed on the log scale up to 21 days to capture the long-lagged effects of heat and cold and to account for short-term harvesting, as done in previous studies ^15^. We then reduced the bi-dimensional temperature-lag response curve to the one-dimensional overall cumulative exposure-response association.

In a second stage, we derived the pooled cumulative exposure-response associations for each cluster type through a multivariate meta-regression model ^40^. We included an indicator of the typology to predict the pooled urban/peri-urban/rural-specific exposure-response curves. To account for specific within-typology variation of spatial and socio-demographic variables, we included the two principal components (PC1 and PC2) summarizing the 10 cluster-level selected variables in the meta-regression model. We assessed the heterogeneity using the likelihood-ratio test and the Cochran Q-test and the *I*^2^ statistic (Table S3). We then predicted the pooled urban/peri-urban/rural temperature-mortality association expressed as a relative risk (RR), with the temperature of minimum mortality (MMT) as reference ^40^. The MMT corresponds to the temperature value for which the temperature-mortality risk is minimum, with days with a mean temperature below the MMT are considered cold and above the MMT are considered hot.

### 2.6 Assessment of the vulnerability factors

To assess vulnerability factors across Switzerland by typology, we applied the same multivariate meta-regression framework used before but separately for each typology and by including each of the 9 vulnerability factors as predictors in univariable models ^34^. In this instance, in each of the univariable meta-analytical models, we separately tested how each predictor modifies the heat and cold-related temperature-mortality association by typology. We predicted the pooled exposure-response curves at the 5^th^ percentile (corresponding to a “low” value) and 95^th^ percentile (a “high” value) value for each of the 9 selected district-specific meta vulnerability factors. Thus, for each typology (urban/peri-urban/rural) we aimed to compare the heat- and cold-related mortality association for the hypothetical high and low levels of the vulnerability factor and subsequently calculated the corresponding p-value between “high” and “low” exposure for each vulnerability factor using the Wald-test. For example, “high” level of exposure to travel time to health care means longer travel time to health care in that specific cluster (corresponding to the 95th percentile), whilst “low” exposure represents short travel time to healthcare for a given cluster by typology (5th percentile). Similarly, exposure to “high” ageing represents a higher ratio of population aged over 65 years compared to the 20-64 age group present in a cluster compared “low” exposure to ageing for a given typology, which has a smaller proportion of people aged over 65 compared to the 20-64 age group, and thus can be characterised as a younger population. We did this for all vulnerability factors. Then, we extracted the RR at the 1^st^ percentile of the temperature distribution for cold and the 99^th^ percentile for heat for each variable with the corresponding 95% confidence interval. Lastly, to ease interpretability, we computed for each vulnerability factor the absolute relative risk difference between “high” and “low” exposure of the RR estimate.

## 3 Results

**Figure 1:**
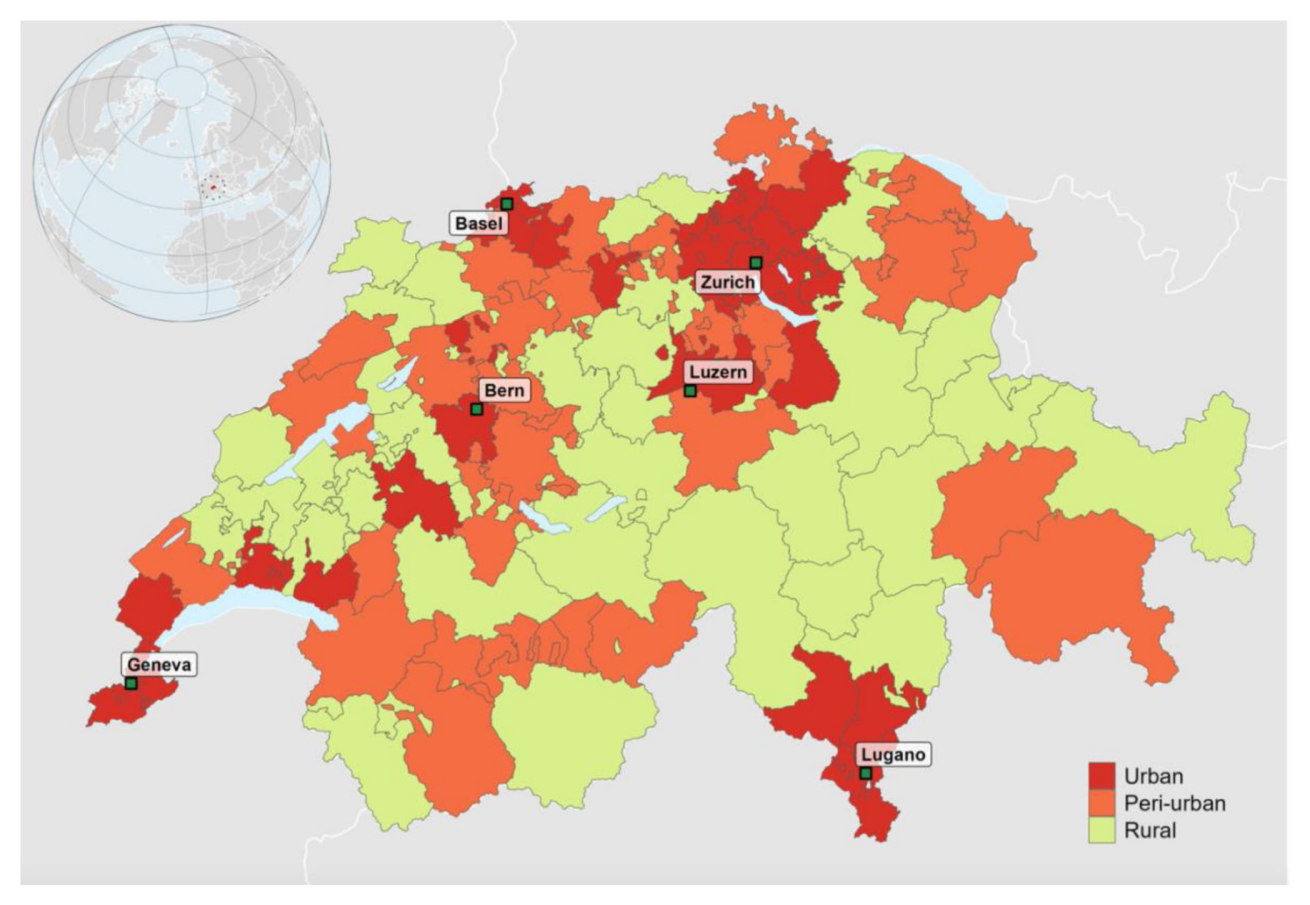
Definition of the 94 clusters based on the Swiss municipalities, which are derived using the Ward-like hierarchical clustering method across the Swiss geography. Urban clusters are indicated in red, peri-urban cluster in orange and rural clusters in yellow, with the main cities indicated in green.

Table 1 provides a summary description of the mortality data, temperature series and the 9 selected vulnerability factors by urban, peri-urban and rural clusters in Switzerland. We analysed 1,775,178 deaths throughout 2,212 municipalities (2,054 aggregated units), covering the full Swiss geography between 1990 and 2017. 48.1% of the deaths occurred in urban clusters (854,077 deaths), followed by peri-urban (577,978) (32.6%) and rural clusters (343,123 (19.3%)) (Figure 1). The urban clusters are mainly located in the North and West of Switzerland, while rural clusters tend to be clustered in Central and East Switzerland, which coincides with the mountainous area of the Swiss Alps (Figure S1-2). Additionally, warmer median temperatures were registered in urban clusters (9.2°C) compared to peri-urban (8.6°C) and rural clusters 7.4°C). Urban clusters also show higher population density compared to rural clusters (1,233 people (interquartile range=854;2,070) versus 226 people (117;288), per km^2^), as well as slightly elevated annual levels of PM_2.5_ (12.4 μg/m^3^(11.4;13.0) versus 9.9μg/m^3^(9.4;10.4)) and shorter time to health care (2.9 minutes (1.8;3.7) versus 6.8 minutes (4.9;11.4)).

**Figure 2:**
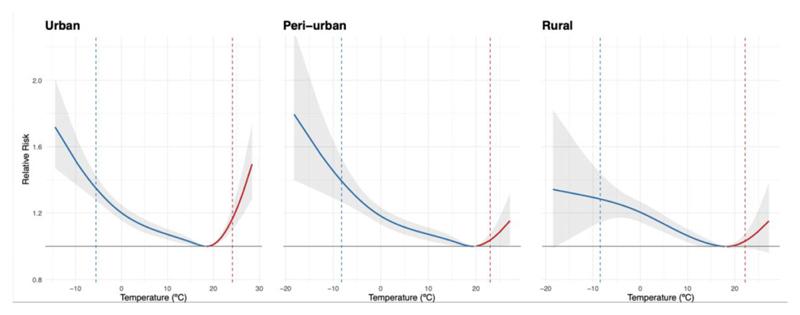
Overall cumulative exposure-response curves in urban, peri-urban and rural clusters in Switzerland between 1990 and 2017. The temperature-mortality association is expressed as relative risk with the corresponding 95% empirical confidence intervals (shaded area), with the temperature of minimum mortality as reference. The blue dashed line represents the 1^st^ percentile and the red line the 99^th^ percentile of the temperature distribution

Figure 2 illustrates the overall cumulative exposure-response curve representing the temperature-mortality association in urban, peri-urban and rural clusters. On average, urban clusters show some evidence for a larger heat-related mortality risk (at the 99^th^ percentile of the temperature distribution) with a RR of 1.17 (95% CI: 1.10;1.24) compared to peri-urban and rural clusters (1.03 (95% CI: 1.00;1.06) and 1.03 (95% CI: 0.99;1.08), respectively). For cold, urban and peri-urban clusters show a similar risk (1.35 (95% CI: 1.28;1.43) and 1.39 (95%CI: 1.27;1.53), respectively), while rural clusters show signs of a slightly lower risk (1.28 (95%CI: 1.14;1.44)), although the confidence intervals partly overlap. There is some evidence for differential patterns of overall non-optimal temperature-mortality association across urban, peri-urban and rural clusters based on the Wald test (p-value = 0.13).

**Figure 3:**
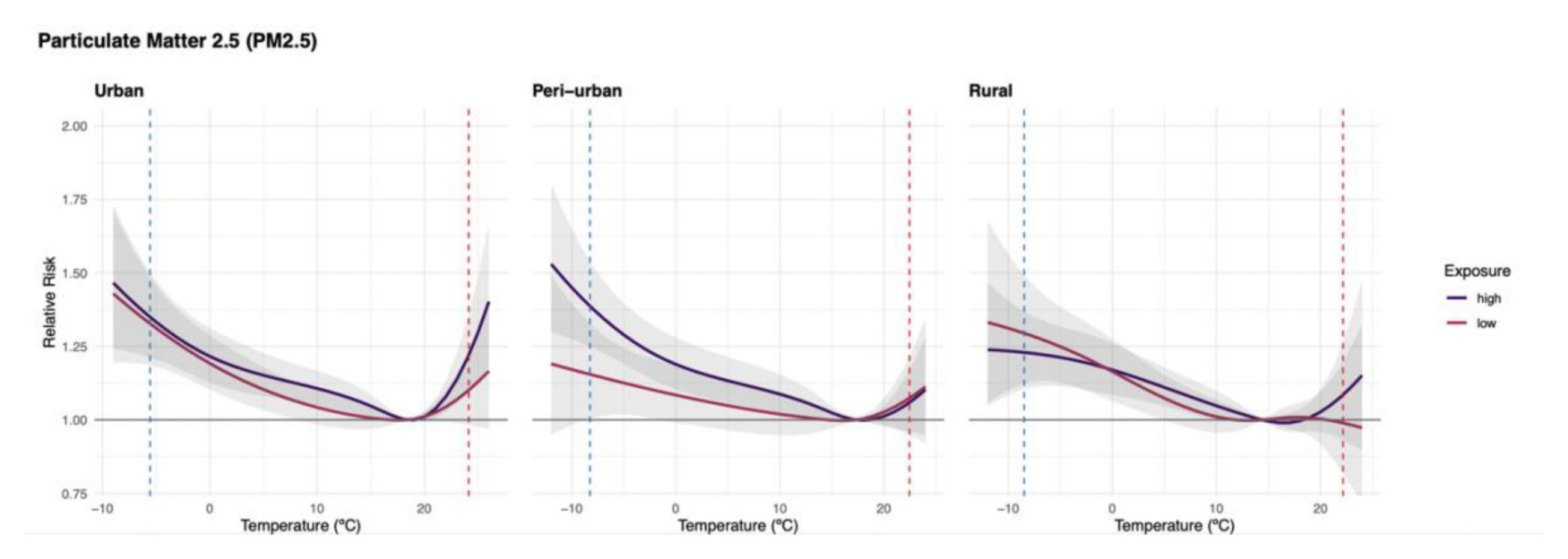
The temperature-mortality association for clusters with exposure to the the 5^th^ percentile of the PM_2.5_ concentration distribution(“low” exposure to PM_2.5_) and the 95^th^ percentile (“high exposure to PM_2.5_) of the urban, peri-urban and rural typologies based on the second-stage univariate-meta-regression model. The association is expressed as relative risk and 95% confidence intervals (shaded area), with the temperature of minimum mortality as reference. The blue dashed line represents the 1^st^ percentile and the red line the 99^th^ percentile of the temperature distribution

For illustrative purposes, Figure 3 shows the temperature-mortality association by typology predicted at high and low levels of annual mean PM_2.5_ concentration (defined as the 95^th^ and 5^th^ percentile, in purple and pink, respectively) using the univariable meta-regression model (i.e., including only PM_2.5_ concentration as predictor). Urban clusters with high annual mean PM_2.5_ concentrations show a larger heat-related mortality risk (1.21 (95%CI:1:10;1.36) indicated with the red vertical dashed line) compared to clusters with low PM_2.5_ (1.09 (95%CI:0.98;1.23)), which is associated with a lower heat-mortality risk. A similar pattern can be observed for rural locations, while for peri-urban clusters no differences were found in the heat tail. Instead, for peri-urban clusters with high PM_2.5_ concentrations we observed a larger cold-mortality risk (1.39 (95%CI:1.25;1.54)) versus 1.15 (95%CI:1:00;1.33)) for low levels of mean annual PM_2.5_ concentration, while similar risks can be observed for urban and rural clusters for cold.

**Figure 4:**
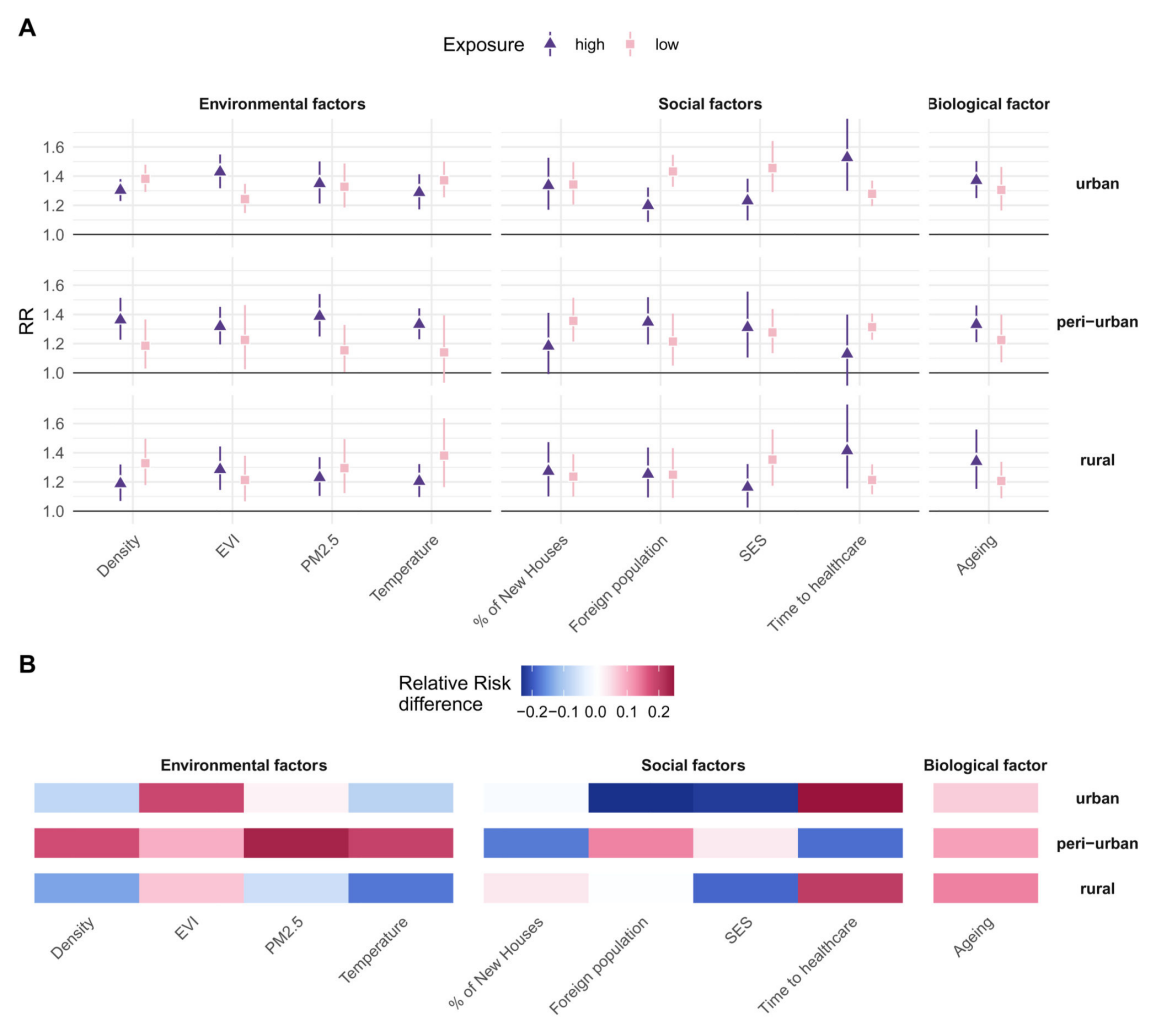
Relative risk for cold (at the 1st percentile, versus temperature of minimum mortality) by low and high exposure to each vulnerability factor. The cold-related relative risk of “low” exposure (5^th^ percentile) is indicated as a pink cube while for “high” exposure (95^th^ percentile) for each vulnerability factor is indicated as a purple triangle together with the corresponding 95% confidence interval (Figure 4A). The absolute Relative Risk difference between “high” and “low” exposure to vulnerability factor is indicated in Figure 4B. A high exposure to a vulnerability factor associated with lower risk is indicated in blue, while a high exposure associated with a higher risk is illustrated in red. High exposure to each vulnerability factor is a high air pollution concentration, high temperature, high proportion of new houses, high ageing index, high proportion of foreign population present, high socio-economic status, long travel time to health care, high population density and high values for EVI for urban, peri-urban and rural clusters.

**Figure 5:**
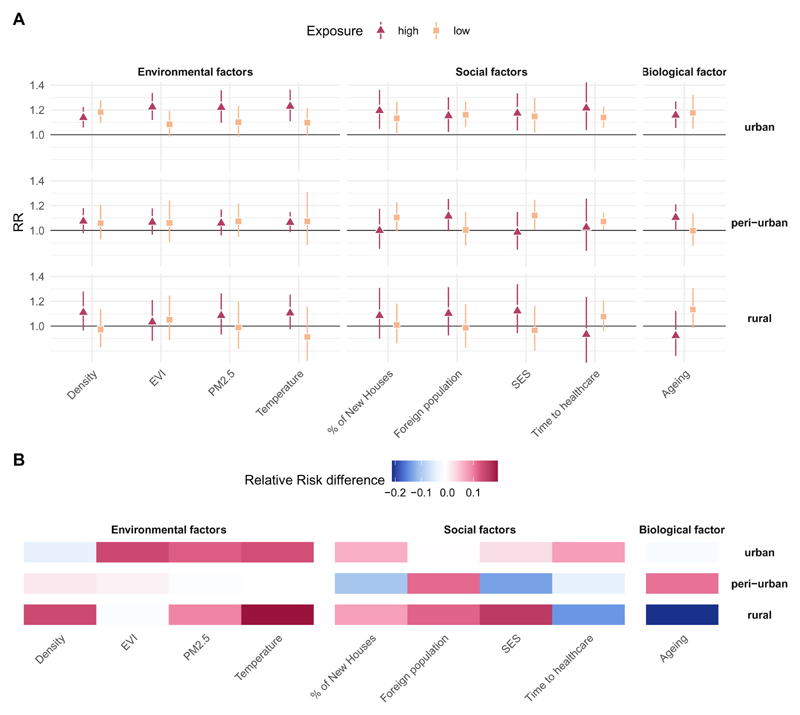
Relative risk for heat (at the 99th percentile, versus temperature of minimum mortality) by low and high exposure for each vulnerability factor. The heat-related relative risk of low exposure (5^th^ percentile) is indicated as an orange cube while the RR for high exposure (95^th^ percentile) is indicated as a red triangle together with the corresponding 95% confidence interval (Figure 5A). The absolute Relative Risk difference between “high” and “low” exposure to vulnerability factor is indicated in Figure 5B. A high exposure to a vulnerability factor associated with lower risk is indicated in blue, while a high exposure associated with a higher risk is illustrated in red. High exposure to each vulnerability factor is a high air pollution concentration, high temperature, high proportion of new houses, high ageing index, high proportion of foreign population present, high socio-economic status, long travel time to health care, high population density and high values for EVI for urban, peri-urban and rural clusters.

Figures 4A and figure 5A illustrate the cold and heat-mortality risks predicted at low (5^th^ percentile) and high (95^th^ percentile) levels for the selected vulnerability factors, respectively. The full exposure-response functions for each vulnerability factor (as shown in Figure 3) are reported in Figure S8-S10 and the complete list of estimates is reported in Table S4-S6. The heat and cold-mortality risks for low exposure to vulnerability factors are indicated with a light pink and orange cube, respectively, while high exposure is indicated with a purple and red triangle, with the corresponding 95% confidence intervals. Figure 4B and figure 5B illustrate the absolute relative risk difference between “high” and “low” exposure to a vulnerability factor. A high exposure to a vulnerability factor associated with lower risk is indicated in blue, while a high risk associated with a higher risk is illustrated in red.

Figure 4 shows that the most influential drivers for cold-related vulnerability across all typologies are social factors while for peri-urban and rural clusters also environmental factors and variables related to urban characteristics are important effect modifiers. High SES and low % of foreign population in urban and rural clusters are associated with a reduction in risk, whilst for peri-urban clusters mixed associations are observed. In urban clusters, high SES is associated with a reduction in risk (1.16 (95%CI1.02;1.32) versus low 1.35 (1.17;1.56)), while long travel time to closest healthcare facility increases the risk for cold (long 1.53 (95%CI:1.30;1.80) versus short 1.28 (95%CI:1.20;1.37)) as well as a large % of foreign population (1.43 (95%CI:1.33;1.55)) versus small % (RR=1.20 (1.09; 1.32). Dissimilar to urban clusters, peri-urban clusters with a high % of foreign population (1.35 (1.20;1.52), versus low 1.21 (95%CI:1.05 ;1.41)) and clusters with a long time to healthcare (1.13 (95%CI:0.91;1.40) versus short 1.31 (95%CI:1.23;1.41)) is negatively associated with cold-mortality risk. Furthermore, stronger effect modification occurs between environmental factors and cold-vulnerability in peri-urban and rural clusters such as annual mean PM_2.5_ concentration, temperature range annual mean temperature. In peri-urban the higher exposure to vulnerability variables yields higher risk (i.e. high temperature (1.33 (95%CI:1.23;1.44) versus low 1.14 (95%CI:0.93;1.39)), while in rural clusters the association is reversed (high temperature 1.38 (95%CI:1.16;1.64), versus low 1.20 (95%CI:11.10;1.32)). In peri-urban clusters also factors such as high population density somewhat increase the risk (1.36 (95%CI:1.23;1.51), versus low 1.19 (95%CI:1.03;1.36)), whilst high density yields lower risk in rural clusters.

Figure 5 shows that the main drivers for heat-related mortality in urban clusters are environmental factors whilst for peri-urban and rural clusters also social factors and biological factors are important drivers of heat vulnerability. In rural clusters, similar to urban clusters, environmental factors such as high PM_2.5_, temperature and temperature range are somewhat associated with a higher risk (i.e. rural clusters with a high annual mean temperature (1.11 (95%CI:0.97;1.25) versus low (0.91 (95%CI:0.72;1.16)) (Figure 5A). Also, social factors and population ageing show evidence for increased heat-related vulnerability. Rural clusters with a high SES show somewhat an increased risk (1.12 (95%CI:0.94;1.34) versus low SES 0.97 (95%CI: 0.80;1.16)), whilst rural clusters with a high proportion of population above 65 years of age show a lower risk to heat-related mortality. The only driver in peri-urban clusters showing some effect modification is a higher SES, which is somewhat associated with a lower risk for heat related-mortality (0.99 (95%CI:85;1.15), versus low SES 1.12(95%CI:1.01;1.25)) and peri-urban clusters with a large proportion of population above 65 years somewhat show an increase in risk (1.10 (95%CI:1.01;1.21)) versus 1.00, (95%CI:0.87;1.14)).

## 4 Discussion

This nationwide study aimed to assess how vulnerability to heat and cold varies across urban, peri-urban and rural clusters, and more importantly, to identify which factors are driving such vulnerability patterns. Our results suggest that urban clusters are at increased vulnerability to non-optimal temperatures, mainly to heat, compared to rural and peri-urban clusters in Switzerland. Therefore, health impacts derived at national or large regional level may be under- or overestimated if ignoring differential vulnerability between urban and rural regions. This may be relevant for the evaluation of historical and future health impacts of climate change. More importantly, our findings challenge the assumption that urban/peri-urban/rural regions share similar vulnerability drivers in terms of characteristics of the population, geographic and socio-economic factors. The main driver for the heat-mortality association across all urban/peri-urban/rural clusters are environmental factors (i.e. temperature and PM_2.5_), however, for peri-urban and rural regions other factors also modify the association such as socio-economic factors as well as population ageing. For cold, across all urban/peri-urban/rural clusters social factors (% of foreign population, SES and travel time to nearest health care facility) modify the cold-mortality association, while for peri-urban and rural clusters also environmental factors and biological factors affected the cold-mortality association, with heterogeneity in the direction of the association between typologies. Although not all identified vulnerability factors such as biological factors are modifiable, this study can help identify vulnerable subpopulations in Switzerland in specific tasks like vulnerability mapping^25,41^. Moreover, future public health adaptation strategies which aim to attenuate heat and cold-related health impacts should account for heterogeneity and implement more tailored interventions according to the local characteristics of the population.

Overall, we observed a larger heat-related mortality risk in urban clusters, followed by peri-urban and then rural clusters, similar to findings from recent assessments^16,32,42–44^, whilst other studies reported larger vulnerability in rural areas ^14,17,18,30,31,33,45,46^. Possibly, these contradictory results could be explained by differences in the baseline health and/or characteristics of the population of urban and rural populations between countries assessed with different baseline characteristics on access to healthcare, population ageing and SES^17,21^. For cold, however, we found some evidence that rural/low-density peri-urban regions yielded lower risks.

We evaluated for the first time the vulnerability factors by different types of regions for both heat- and cold-related mortality in a nationwide study setting. Thus far, previous assessments have primarily aimed to identify heat-related vulnerability factors in single ^19,20,25^ or multi-location analyses ^21,47^ while disregarding the potential heterogeneity in vulnerability and associated drivers by type of area. In this study, we applied the novel extended two-stage design recently developed and we observed substantial differences in vulnerability factors between types of areas driving the temperature-mortality association^34^. could be in part driven by the urban heat island effect as our findings suggest that environmental vulnerability related to urban characteristics (high mean temperature and high PM_2.5_ concentrations) were associated with increased vulnerability which is in accordance with literature^32,48,49^. However, we cannot disentangle the contribution of the UHI and/or any of the other drivers due to the complex correlation between them and the lack of specific UHI metrics^50^. Regarding social vulnerability, previous assessments found that low SES, social isolation and population ageing could increase vulnerability in urban areas, for the former we observed good evidence for effect modification for both heat (peri-urban clusters) and cold (urban and rural clusters), where higher SES is associated with a reduction in risk^13,19,21,25,44,51–53^. Despite increased risk for heat mortality with lower SES, we did not observe patterns in urban heat exposure and climate injustice between clusters, which usually is present on an intra-city level^54–56^. For peri-urban clusters, where besides environmental factors, also social (i.e., SES, travel time to healthcare) and biological factors (i.e., ageing) were found to be important effect modifiers. In contrast to many previous studies, we did not observe evidence for greenness as an effect modifier for the heat-mortality risk ^20,21,25,52,57,58^. This may be explained by the limited variability in the EVI values across urban and peri-urban clusters in Switzerland (IQR of 0.41-0.45 and 0.42-0.49, respectively). Although for urban clusters we found a negative association between greenness and temperature, for peri-urban and rural clusters, temperature was positively correlated with greenness (possibly since the level of greenness at high altitude in the Alps is missing, therefore, reducing the spread of EVI) (Fig S1-2 and S6), illustrating the limitation of using EVI as a universal indicator for greenness without making regional distinctions.

Unlike heat vulnerability, evidence on cold-related vulnerability factors remains limited and inconclusive in the literature^16 21^. Our findings suggest that cold-related vulnerability in urban clusters was mainly driven by socio-economic factors (e.g., long travel time to time health care, % of foreign population and SES) as well as population density, consistent with previous studies ^21,22^, while more relevant drivers were identified in peri-urban and rural clusters such as % of new houses, PM_2.5_, ageing and population density. To note, although many previous studies found that low-housing quality exacerbates the risk of heat ^22,52^, this is one of the more recent studies that also report housing as an effect modifier for cold-related mortality, particularly in peri-urban regions ^59,60^. It has been hypothesised that the reason for the existing inconclusive findings or complex patterns on cold vulnerability might be due to the more complex pathways of how cold exposure can affect health (i.e., infectious diseases, public health interventions). Future research should aim to study cold-related vulnerability factors and clarify the links between factors and mechanisms driving increased risks.

A result worth highlighting is the heterogeneity in the direction of effect modifiers of the heat and cold-mortality association by urban/peri-urban/rural clusters. We found that environmental factors (i.e. PM_2.5_ concentration and mean annual temperature), as well as population density, are negatively associated with cold-related mortality in rural clusters while positively associated in peri-urban clusters. Meaning that peri-urban clusters that are more similar to urban clusters have higher risks for cold-related mortality than peri-urban clusters which are more similar to rural clusters. Moreover, rural clusters with higher temperatures, higher PM_2.5_ concentrations and higher population density that are generally associated with increased urbanisation have a lower risk than rural clusters with low temperatures and low population density. Therefore, we believe that for cold-related mortality the lowest risk can be observed in rural/low-density peri-urban areas, a finding shared by a recent study that observed this association for heat^17^. For heat, however, we only observed increased vulnerability for urban clusters, which might be due to the urban heat island, while in peri-urban and rural no differences were found, possibly because outside of the main cities, Switzerland is very sparsely populated (Figure S6).

This study has several strengths. First, we used advanced statistical methods recently developed in climate epidemiology to maximize the power of the available data and increase the precision of our estimates and the reliability of our conclusions. In particular, we applied the novel case time series design which allowed us to use temperature and mortality data at a high resolution and thereby increase the precision of the risk estimates ^38^. We used DLNMs to account for the complexity of the temperature-mortality association, in terms of potential non-linearities and delayed effects up to 21 days. To pool the risks and assess the proposed vulnerability factors, we then applied a complex meta-analytical model which properly accounted for the hierarchical structure and heterogeneity of the risks (Sera et al., 2020). Finally, to assess the effect modification of vulnerability factors we applied a novel extended second-stage time series which allowed us to test vulnerability factors in the complex non-linear mixed meta regression which has not been applied to nationwide data before^34^. Then, to assess vulnerability factors in both urban and rural areas we used gridded climate datasets which allowed us to assign temperature exposure at municipality level, which is considered an unprecedented high resolution in many ecological studies investigating temperature and mortality^36^. Lastly, using the Ward-like hierarchical clustering with geographical constraints ^37^, we defined ad hoc clusters of municipalities with similar characteristics, as an alternative to the administrative district boundaries (i.e., an upper geographical unit above the municipality) defined by the BFS (BFS, 2021). The Swiss orography and population distribution are highly heterogeneous, with large variation in demographic and environmental variables within administrative clusters and thus the effect modification of vulnerability factors by cluster typology can be diluted if heterogenous municipalities are grouped in the same cluster.

Some limitations should be acknowledged. First, our findings suggest vulnerability patterns according to levels of specific vulnerability factors but do not remove effects from correlated variables. That is, risks at different levels of the vulnerability factor were derived using univariate models, thus, not accounting for other (correlated) factors that might partly explain differences in the temperature-mortality association within typologies (i.e., by the UHI). Second, the low statistical power in rural clusters hindered the assessment of vulnerability factors. Additionally, we observed limited variability for some effect modifiers by urban/peri-urban/rural typologies, which could have limited the power to detect effect modification by variable. Then, we did not include humidity, influenza and air pollution concentrations as confounding variables, as we believe that their impact would be, if present, minimal, as their role as an confounding variable remain debated ^63–65^. Lastly, this is an ecological assessment conducted at the municipality level. Thus, our results would not necessarily correlate with evidence on vulnerability factors driving differences at a finer resolution within municipalities (i.e., neighbourhood).

## 5 Conclusion

Our findings suggest larger temperature vulnerability in urban clusters, particularly for heat compared to rural regions, while cold-related vulnerability was similar across typologies. More importantly, this study has shown that drivers of temperature vulnerability can considerably vary by urban-rural typology in Switzerland. Therefore, future public health adaptation strategies aimed at mitigating the adverse impacts of climate change on population health should consider tailored interventions according to the characteristics of the target population.

## Supplementary Material

Supplementary file

## Figures and Tables

**Table 1:** Number of clusters, municipalities, total all-cause deaths and average daily mean temperature between 1990 and 2017 and range and mean value and inter quartile range of the selected vulnerability factors (see Table S1 for the complete list of variables, source and description) by type of cluster in Switzerland.

	Urban	Peri-urban	Rural
**Clusters (N)**	26 (27.6%)	31 (33.0%)	37 (39.4%)
**Municipalities (N)**	557 (27.1%)	770 (37.5%)	727 (35.4%)
**All-cause deaths (N)**	854,077 (48.1%)	577,978 (32.6%)	343,123
			
**Temperature (°C)**	9.2 (3.5; 15.3)	8.6 (2.8; 14.4)	7.4 (2.6; 14.1)
**SES index**	53.4 (49.0; 55.4)	47.2 (44.9; 49.9)	44.6 (41.5; 47.9)
**Ageing index**	50.1 (46.2; 53.8)	48.8 (47.8; 50.9)	48.2 (45.2; 51.2)
**New houses (%)**	5.2 (4.0; 6.5)	6.3 (4.5; 8.0)	5.9 (3.4; 8.2)
**Time to healthcare (minutes)**	2.9 (1.8; 3.7)	3.6 (3.2; 6.1)	6.8 (4.9; 11.4)
**PM_2.5_ (μg/m^3^)**	12.4 (11.4; 13.0)	10.5 (9.9; 11.6)	9.9 (9.4; 10.4)
**Enhanced vegetation index (EVI)**	0.45 (0.41; 0.45)	0.46 (0.42; 0.49)	0.47 (0.44; 0.50)
**Density (per km^2^)**	1,233 (854; 2,070)	640 (441; 831)	226 (117; 288)
**Foreign population (%)**	23.4 (20.7; 27.3)	20.1 (15.9; 21.3)	14.3 (11.1; 17.9)
