# Supplementary material for: Exploring vulnerability to heat and cold across urban and rural populations in Switzerland
Source: Environ Res Health. Author manuscript; Available in PMC 2023 Jun 1. (PMC7614344; doi:10.1088/2752-5309/acab78)
Supplement: Supplementary file [file EMS164575-supplement-Supplementary_file.docx]

**Exploring vulnerability to heat and cold: mortality risks across urban and rural populations in Switzerland.**

**Authors: Evan de Schrijver^1-3^, Dominic Royé^4,5^, Antonio Gasparrini^6-8^, Oscar H. Franco^1^, Ana M. Vicedo-Cabrera^1,2^.**

1. Institute of Social and Preventive Medicine (ISPM), University of Bern, Bern, Switzerland
2. Oeschger Center for Climate Change Research (OCCR), University of Bern, Bern, Switzerland.

3. Graduate school of Health Sciences (GHS), University of Bern, Bern, Switzerland

4. Department of Geography, University of Santiago de Compostela, Santiago de Compostela, Spain

5. CIBER of Epidemiology and Public Health (CIBERESP), Spain

6. Centre on Climate Change and Planetary Health, London School of Hygiene & Tropical Medicine, London (LSHTM), London, United Kingdom

7. Department of Public Health, Environments and Society, London School of Hygiene & Tropical Medicine, London United Kingdom

8. Centre for Statistical Methodology, London School of Hygiene & Tropical Medicine, London United Kingdom

**Table of content**

**Methods**

**Methods S1:** Ward-like hierarchical clustering algorithm

**Tables**

**Table S1:** Definition and sources of the selected vulnerability factors

**Table S2:** First stage sensitivity analysis

**Table S3:** Model output of the second stage mixmeta regression and model selection.

**Table S4.** Cold-related relative risks for high and low levels of each vulnerability factor by urban, peri-urban and rural area in Switzerland

**Table S5.** Heat-related relative risks for high and low levels of each vulnerability factor by urban, peri-urban and rural area in Switzerland

**Table S6**. Results of the Wald-test of the comparison between the exposure-response curves derived for high and low levels of each vulnerability factor for urban, peri-urban and rural areas in Switzerland.

**Figures**

**Figure S1.** Population distribution for Switzerland at a 1x1 km resolution

**Figure S2.** Elevation for Switzerland at a 1x1 km resolution

**Figure S3.** Spatial distribution of 42 collected variables at municipality level resolution in Switzerland

**Figure S4.** Correlation matrix for all 42 collected vulnerability factors at district level

**Figure S5.** Coordinates of Principal Components

**Figure S6**. Correlation between the selected vulnerability factors by urban (A), peri-urban (B) and rural (C) clusters

**Figure S7*.*** Mixing alpha between Matrix D_0_ and D_1_ for different values in 94 clusters.

**Figure S8**. Exposure-response curves for high (95^th^ percentile) and low (5^th^ percentile) levels of each vulnerability factor in urban districts in Switzerland.

**Figure S9**. Exposure response curves for high (95^th^ percentile) and low (5^th^ percentile) levels of each vulnerability factor in peri-urban districts in Switzerland.

**Figure S10**. Exposure response curves for high (95^th^ percentile) and low (5^th^ percentile) levels of each vulnerability factor in rural districts in Switzerland.

**References**

**Methods S1: Ward-like hierarchical clustering algorithm**

The Ward-like algorithm is a constrained hierarchical clustering algorithm that aims to optimize the convex combination Dα = (1−α) D_0_ + αD_1_, using two dissimilarity matrices (D_0_ and D_1_) and a mixing parameter α∈[0;1] at a municipality level resolution (Chavent et al., 2018). Matrix D_0_ = [d_0_,_ij_] is constructed based on the Manhattan distance matrix of 2,054 municipalities based on six selected spatial and demographic variables (i.e. temperature, temperature range, ageing index, social index, density and impervious surfaces). Matrix D_1_ = [d_1_,_ij_], which represented the spatial constraint space which was based on the geographical distance between the coordinates of the corresponding municipalities centroids. The inclusion of alpha assigns the relative importance of D_0_ and D_1_. When α = 0 (resp. α = 1), the hierarchical clustering is only based on matrix D_0_ (resp. = D_1_). We set α = 0.7, as this value increased spatial homogeneity without substantially reducing the quality of the solution of matrix D_0_, or the spatial-demographic variables(Figure S2). The mixed pseudo-inertia of cluster $C_{k}^{\alpha}$ is:

$$I_{\alpha}\left( C_{k}^{\alpha} \right)=(1-\alpha)\sum_{i\in C_{k}^{\alpha}} \sum_{j\in C_{k}^{\alpha}} \frac{w_{i}w_{j}}{2\mu_{k}^{\alpha}}d_{0,ij}^{2}+^{\alpha}\sum_{i\in C_{k}^{\alpha}} \sum_{j\in C_{k}^{\alpha}} \frac{w_{i}w_{j}}{2\mu_{k}^{\alpha}}d_{1,ij}^{2}$$

where $\mu_{k}^{\alpha}=\sum_{i\in C_{k}^{\alpha}} w_{i}$ is the weight of $C_{k}^{\alpha};d_{0;ij}\text{ and }d_{1;ij}$ are the normalized dissimilarity between observations *i* and *j* in D_0_ and D_1_, respectively. Subsequently we created a new agglomerative level consisting of *k*=94 clusters, which was based on municipalities that were both similar- and proximal to each other and had a minimum of 1,000 deaths. As such, we aggregated the municipalities that the new cluster had a minimum mixed-within cluster inertia, or were most similar to each other within a cluster:

$$W_{\alpha}\left( \mathcal{P}_{K}^{\alpha} \right)=\sum_{k=1}^{K} I_{\alpha}\left( \mathcal{C}_{k}^{\alpha} \right)$$

Once we redefined the new cluster boundaries, we then assigned a definition of “urban”, “peri-urban” or “rural” to the cluster depending on the relative number of people residing inside an “urban” or “rural” of the municipalities (defined by the BFS (within the newly created higher agglomerative cluster (BFS,2021)). When < 50% of the population lived in urban municipalities of the corresponding cluster we considered the cluster rural, when 50-80% resided in an urban municipality it was considered peri-urban and when >80% of the population resided in an urban municipality we considered the new cluster to be an urban region.

**Table S1.** Definition and sources for the selected vulnerability factors

| Variable | Definition | Year | Source |
| --- | --- | --- | --- |
| Foreign population | The proportion of permanent foreign population in the total permanent resident population, as well as the total number of permanent foreign resident population. | 2010 | Bureau of Federal Statistics |
| % > 65 | % of people aged above 65 | 2000 | Bureau of Federal Statistics |
| Deaths | The number of deaths per 1,000 persons in the mean permanent resident population and the total number of deaths.  Number of deaths in a given calendar year per 1000 persons in the mean permanent resident population in the middle of the year. | 2010 | Bureau of Federal Statistics |
| Crime rate | The number of crimes committed per 1,000 persons in the mean permanent resident population. | 2010 | Bureau of Federal Statistics |
| Population density | The number of people residing in a municipality according to the Columbia NASA | 2015 | Socioeconomic Data and Applications Center SEDAC, Columbia Universtiy |
| One family house | The percentage of single-family houses in the total building stock.  In order to facilitate the application of the data, missing or incorrect values ​​of the GWS were used statistically. In the case of small-scale evaluations, it cannot be ruled out that these additions lead to deviations that do not correspond to reality.  The building and housing statistics (GWS) refer to all buildings with residential use in Switzerland on December 31 of the reference year. They include purely residential buildings (single-family houses, multi-family houses), residential buildings with secondary use (e.g. residential buildings with commercial premises, farmhouses, etc.) as well as buildings with partial residential use (e.g. administrative buildings or school buildings with waiting apartments, but also hotels, hospitals, homes, etc.).  Single-family houses are purely residential buildings with one apartment.  Buildings are permanent structures, firmly connected to the ground, which serve residential purposes or purposes of work, education, culture or sport. In the case of semi-detached, group and row houses, each building counts as independent if it has its own access from the outside and if there is a vertical load-bearing separating wall between the buildings that extends from the ground floor to the roof. | 2010 |  |
| New houses | The number of new homes built in the year in question per 1000 inhabitants and the total number of new homes built in the year in question. Newly created apartments due to renovations are not counted.  The term apartment is understood to mean the entirety of the rooms that form a structural unit and have their own access either from the outside or from a common area within the building (staircase). An apartment in the sense of statistics has a cooking facility (kitchen or kitchenette). A single-family house consists of one apartment; Single-family houses with separate apartments are recorded as multi-family houses. All apartments are counted regardless of whether the apartment is intended for private or collective households. | 2010 | Bureau of Federal Statistics |
| 3-4 rooms | the percentage of 3-4 room apartments in the total housing stock.  Rooms are living spaces such as living rooms, bedrooms, children's rooms, etc., which together form an apartment. The kitchen, bathrooms, showers, toilets, reduits, corridors, half rooms, verandas and additional separate living rooms outside the apartment are not counted.  To make the application of the data easier, missing or incorrect values of the GWS were used statistically. In the case of small-scale evaluations, it cannot be ruled out that these additions lead to deviations that do not correspond to reality. | 2010 | Bureau of Federal Statistics |
| Net income | The average taxable income of natural persons in a municipality, based on the permanent resident population living there. All residents - including those who have no income and children - are included. The assessment basis is the relevant taxable income for calculating the federal tax. | 2010 | Bureau of Federal Statistics |
| Live births | the number of live births per 1000 persons in the mean permanent resident population and the total number of live births.  Births of living children, i.e. children who die after they have left the womb completely (body, head and limbs) show signs of life (breathing or heartbeat). | 2010 | Bureau of Federal Statistics |
| PM2.5 | The PM2.5 grids consist of concentrations (micrograms per cubic meter) of ground-level fine particulate matter (PM2.5) – dust and sea-salt removed – per 0.01 degree grid cells for each of the nineteen years between 1998 and 2016. | July - 2010 | Van Donkerlaar et al., 2018 |
| Mean trees | Number of trees per municipality divided by the number of grid cells included | July-2010 | MODIS, Google Earth Engine |
| Access to city | the time required for individuals to reach their most accessible city, |  | Weiss et al.,2018 |
| Access to healthcare | travel time to hospitals and clinics |  | Weiss et al., 2020 |
| Impervious surfaces | Global Man-made Impervious Surface (GMIS) Dataset From Landsat.  To provide high spatial resolution estimates of global man-made imperviousness for the target year 2010, derived from global 30m Landsat satellite data and a companion dataset to the Global Human Built-up And Settlement Extent (HBASE) dataset. |  | Socioeconomic Data and Applications Center SEDAC, Columbia Universtiy |
| NDVI (Normalised Difference Vegetation Index) | Mean NDVI for July 2010 | July- 2010 | MODIS, Google Earth Engine |
| Albedo | Mean Albedo for July 2010 | July- 2010 | MODIS, Google Earth Engine |
| EVI (Enhanced Vegetation Index) | Mean EVI for July 2010 | July-2010 | MODIS, Google Earth Engine |
| Wet area | The percentage of water and wet areas (water, glaciers, wet areas) of the total area. The exact definition of the land cover is based on the nomenclature NOLC04.  Water areas of standing and flowing waters, wet areas covered with reeds and other moisture-loving plants as well as glacier and firn areas. | 2004-2009 | Bureau of Federal Statistics |
| Artificial area | the percentage of artificially created areas of the total area. The exact definition of the land cover is based on the nomenclature NOLC04.  Artificially created, partially sealed and built over with civil engineering, partially natural areas with garden or park-like planting, including lawns and trees on such areas. | 2004-2009 | Bureau of Federal Statistics |
| Building area | the percentage of building created areas of the total area. The exact definition of the land cover is based on the nomenclature NOLC04.  Building created, partially sealed and built over with civil engineering, partially natural areas with garden or park-like planting, including lawns and trees on such areas. | 2004-2009 | Bureau of Federal Statistics |
| Constructed area | the proportion of sealed areas (paved areas, buildings and greenhouses) in relation to the total area. The exact definition of the land cover is based on the nomenclature NOLC04.  Artificially created surfaces that are completely or partially impermeable to rainwater. This includes completely sealed surfaces such as buildings, asphalt and concrete floors, as well as partially sealed surfaces such as gravel, marbled floors and floors covered with stones or slabs. In the area statistics, the sealed areas are calculated from the sum of the land cover categories paved areas, buildings and greenhouses. | 2004-2009 | Bureau of Federal Statistics |
| Farm area | the percentage of agricultural areas in the total of the total area. The exact definition of land uses is based on the NOAS 2004 standard nomenclature. | 2004-2009 | Bureau of Federal Statistics |
| Industrial area | the percentage of the industrial and commercial area in the total of the total area. The industrial and commercial area is part of the settlement area. The exact definition of land uses is based on the NOAS 2004 standard nomenclature.  According to the area statistics, the industrial area includes the area and surrounding areas of buildings with industrial or commercial use. In addition to the actual industrial production facilities, warehouses and storage areas, distribution centers, some military buildings (army motor vehicle parks, armories), sawmills, carpenters' shops, construction companies, car cemeteries and parking areas for car import companies or goods transshipment points also belong to the industrial and commercial area. The turnaround also includes silos, oil tanks, conveyor systems and electrical installations, lawns, ornamental gardens, parking spaces, paths, driveways and industrial tracks as well as forest coverings, bushes and shrubs within the area. The industrial area is part of the settlement area. | 2004-2009 | Bureau of Federal Statistics |
| Settlement area | The percentage of the settlement area in relation to the total of the total area. The exact definition of land uses is based on the NOAS 2004 standard nomenclature.  According to the area statistics, they include all areas and facilities that are used for living, traffic, production (excluding agriculture and forestry), trade and services, supply and disposal as well as recreation. The "special settlement areas" include the supply and disposal systems (energy, wastewater treatment, rubbish, etc.), mining areas, landfills, construction sites and fallow land, as well as buildings on such areas. | 2004-2009 | Bureau of Federal Statistics |
| Traffic area | The percentage of traffic areas in the total of the total area. The traffic area is part of the settlement area. The exact definition of land uses is based on the NOAS 2004 standard nomenclature.  According to the area statistics, the traffic area is the land that is used for public or private rail traffic used for commercial purposes, for public roads, above-ground pipelines for the transport of heating oil and other liquids, airports, installations for the telecommunications system, etc. This also includes Land used for offices and other service buildings and facilities involved in the transport sector. Examples of this are train stations, airport buildings, warehouses for equipment and repair shops, sidewalks, grass-covered embankments on the edge of the railway lines, windbreaks along the roads, noise abatement facilities around airports and other land that is needed in accordance with national practice to provide the appropriate infrastructure. Waterways, on the other hand, do not fall into this category, but the area under water does. The traffic area is part of the settlement area. | 2004-2009 | Bureau of Federal Statistics |
| Unproductive area | The percentage of the unproductive area in the total of the total area. Unproductive areas are essentially bodies of water, glaciers, rocks or other areas that are not used by humans for production or settlement. The exact definition of land uses is based on the NOAS 2004 standard nomenclature.  The unproductive areas according to the Swiss land use statistics include standing water, rivers, unproductive vegetation and areas without vegetation (rock, sand, scree, glaciers and firn). | 2004-2009 | Bureau of Federal Statistics |
| Wooded area | The percentage of the planted area in relation to the total of the total area. The exact definition of land uses is based on the NOAS 2004 standard nomenclature.  Areas overgrown with trees or shrub species that form shrubbery. In the area statistics, the forested areas correspond to the sum of forests and trees. | 2004-2009 | Bureau of Federal Statistics |
| 1 person household | The percentage of households with one person in relation to the total of private households.  A group of people who usually live together, i.e. share a common apartment. The households are divided into private households (which can also include only one person) and collective households. | 2012 | Bureau of Federal Statistics |
| 2 person household | The percentage of households with two persons in relation to the total of private households. | 2012 | Bureau of Federal Statistics |
| 3 person household | The percentage of households with three person in relation to the total of private households. | 2012 | Bureau of Federal Statistics |
| 4 person household | The percentage of households with four person in relation to the total of private households. | 2012 | Bureau of Federal Statistics |
| 5 person household | The percentage of households with five person in relation to the total of private households. | 2012 | Bureau of Federal Statistics |
| Mean household size | The mean number of persons in relation to the total of private households. | 2012 | Bureau of Federal Statistics |
| Tertiary sector | represents the number of people employed in the 3rd economic sector. | 2012 | Bureau of Federal Statistics |
| Individualisation index | The individualization index stands for the degree of deviation from the bourgeois-traditional life model. Individualized forms of life such as single households or the employment of mothers are recorded on the basis of household type and family model. The calculation formula is: Individualization index = 3 EPH + 1.2 WG + 2.5 FOK + 3 MER - 1.5 TBM EPH = one-person households (30 to 50 year olds) Household type: one-person households WG = shared apartments (30 to 50 year olds) Household type: Non-family households with relatives, no other persons, non-family households with relatives and other persons, households of unrelated persons MER = Working mothers (25 to 44 year olds) Labor market status of women in households with children: full-time workers, part-time workers with one or more positions FOK = Women without children (35 to 44-year-olds) women in households without children TBM = traditional middle-class family model Families with children under 16 years of age with labor market status: full-time employment (father), as well as inactive persons and household (mother) for more information on this index In the publication Sociocultural Differences in Switzerland. |  | Bureau of Federal Statistics |
| Linguistic integration | the percentage of the population that does not specify any of the four national languages (German, French, Italian and Romansh) as their main language.  The main language is the language in which the respondents think according to their statements or which they speak best. | 2000 | Bureau of Federal Statistics Bureau of Federal Statistics |
| Social index | the status index as a measure of the social status of the population. The status index is calculated as a weighted sum of six indicators. Three of the six indicators measure the strength of the high status proportion of the population (or the upper class) in a location.  The corresponding indicators are "tertiary education", "top management / liberal professions" and "high income". They flow into the index as positive values. The three other indicators measure the proportion of the population with a low status (or the lower class) in a location. These include the indicators "primary education", "low-status occupations" and "low income", which are included in the index as negative values. This means that the status index of a spatial unit is higher, the larger the proportion of the population with high status characteristics and the smaller the proportion of the population with low status characteristics. The value 50 corresponds to the average for Switzerland.  The calculation is as follows:  Status index = 2.5 • TER - 2 • PRI + OMF - NST + 4 • HEK - 2 • NEK  TER = tertiary education (over 25s)  PRI = primary education (over 25s)  OMF = upper management & liberal professions (employed)  NST = low-status occupations (employed)  HEK = high income (taxpayers)  NEK = low income (taxpayers) | 2000 | Bureau of Federal Statistics |
| Commuters | The balance of inbound and outbound commuters per 100 employees and schoolchildren / students and the difference between inbound and outbound commuters (commuter balance).  Employed person aged 15 and over who has a fixed place of work outside of their home. Commuters therefore do not include those who work at home or those who do not have a fixed place of work (e.g. representatives). | 2000 | Bureau of Federal Statistics |
| Ageing index | Ratio of 65-year-olds and older to 20- to 64-year-olds, i.e. the ratio of the number of people at an age at which one is generally no longer economically active to the number of people of working age | 2000 | Bureau of Federal Statistics |
| Mean Temperature | Daily mean temperature derived at municipality level using a 2km-resolution Gridded Climate Datasets | 1990- 2017 | MetteoSwiss |
| Temperature range | Mean Inter Quartile Range at municipality level using a 2km-resolution Gridded Climate Datasets | 1990-2017 | MetteoSwiss |

**Table S2**. First stage sensitivity analysis based on the overall mean qAIC

| **Exposure response Specifications** | **Lag response specification** | **Lag (days)** | **qAIC** |
| --- | --- | --- | --- |
| Quadratic B-Spline knots (10^th,^ 75^th,^ 90^th)^ | 3 knots | 21 | 66,485 |
| Quadratic B-Spline knots (20^th,^ 50^th,^ 90^th)^ | 3 knots | 21 | 66,487 |
| Quadratic B-Spline knots (10^th,^ 30^th,^ 50^th^,70^th^,90^th)^ | 3 knots | 21 | 66,496 |
| Quadratic B-Spline knots (10^th,^ 75^th,^ 90^th)^ | 2 knots | 10 | 66,587 |
| Quadratic B-Spline knots (10^th,^ 75^th,^ 90^th)^ | 1 knot | 5 | 66,634 |
| Quadratic B-Spline knots (10^th,^ 75^th,^ 90^th)^ | 1 knot | 3 | 66,656 |

**Table S3**. Second-stage meta-regression model. The significance test for the predictors was derived using the Wald-test (p-value), the multivariate Cochran Q-test for heterogeneity (p-value), and *I*^2^ statistic (%) in the different multivariate meta-regression models. The model selection was in part based on the Akaike Information Criteria (AIC) while also taking the Wald-test, I^2^ and Q-test into consideration. Underneath we show results for the crude model and the overall model adjusted by type of area (i.e. urban, peri-urban and rural), PC1 and PC2 (with and without interaction). PC1 and PC2 were derived using the Principal Components Correlation (Figure S4) by type of area.

| **Model** | AIC | I^2^ | Cochran Q-test | Type | PC1 | PC2 | Type: PC1 | Type: PC2 |
| --- | --- | --- | --- | --- | --- | --- | --- | --- |
| **Crude model (no variables)** | 322 | 3.2% | 0.32 |  |  |  |  |  |
| + Type | 332 | 3.1% | 0.32 | 0.28 |  |  |  |  |
| + PC1 + PC2 | 337 | 4.1 % | 0.27 | X | 0.47 | 0.99 |  |  |
| **+ Type + PC1 + PC2** | **345** | **3.1%** | **0.32** | **0.13** | **0.38** | **0.68** |  |  |
| + Type + PC1 + PC2 +  Interaction (type*PC1) +  interaction (type*PC2) | 360 | 2.1% | 0.38 | 0.12 | 0.35 | 0.58 | 0.15 | 0.40 |

**Table S4.** Cold-related mortality risks (relative risk (RR) and 95% confidence interval (CI)) for high (95^th^ percentile) and low (5^th^ percentile) levels of each vulnerability factor by urban, peri-urban and rural area in Switzerland. Cold-related mortality risk corresponds to the RR at the 1st percentile versus the temperature of minimum mortality.

|  | **Urban** | | **Peri-urban** | | **Rural** | |
| --- | --- | --- | --- | --- | --- | --- |
| **Variable** | **RR low (95%CI)** | **RR high (95%CI)** | **RR low (95%CI)** | **RR high (95%CI)** | **RR low (95%CI)** | **RR high (95%CI)** |
| **Ageing** | 1.30 (1.16;1.46) | 1.37 (1.25;1.50) | 1.22 (1.07;1.40) | 1.33 (1.21;1.46) | 1.21 (1.09;1.34) | 1.34 (1.15; 1.55) |
| **Density** | 1.38 (1.29;1.48) | 1.30 (1.23;1.38) | 1.19 (1.03;1.36) | 1.36 (1.23;1.51) | 1.33 (1.18;1.50) | 1.19 (1.07;1.32) |
| **% of new houses** | 1.34 (1.21;1.50) | 1.34 (1.17;1.53) | 1.36 (1.21;1.51) | 1.18 (0.99;1.41) | 1.24 (1.10;1.40) | 1.27 (1.10;1.47) |
| **Social index** | 1.46 (1.29;1.64) | 1.23 (1.10;1.38) | 1.28 (1.13;1.44) | 1.31 (1.11;1.55) | 1.35 (1.17;1.56) | 1.16 (1.02;1.32) |
| **PM2.5** | 1.33 (1.19;1.49) | 1.35 (1.21;1.50) | 1.15 (1.00;1.33) | 1.39 (1.25;1.54) | 1.29 (1.12;1.49) | 1.23 (1.10;1.37) |
| **Time to health care** | 1.28 (1.20;1.37) | 1.53 (1.30;1.80) | 1.31 (1.23;1.41) | 1.13 (0.91;1.40) | 1.21 (1.12;1.32) | 1.41 (1.16;1.73) |
| **Impervious surfaces** | 1.42 (1.31;1.53) | 1.27 (1.18;1.36) | 1.24 (1.09;1.41) | 1.33 (1.19;1.49) | 1.31 (1.16;1.49) | 1.20 (1.06;1.34) |
| **EVI** | 1.24 (1.15;1.35) | 1.43 (1.32;1.55) | 1.22 (1.02;1.46) | 1.32 (1.19;1.45) | 1.21 (1.07;1.38) | 1.29 (1.15;1.44) |
| **Wet bodies** | 1.33 (1.21;1.48) | 1.34 (1.18;1.54) | 1.32 (1.21;1.44) | 1.18 (0.97;1.45) | 1.22 (1.13;1.33) | 1.32 (1.17;1.50) |
| **Loneliness** | 1.36 (1.22;1.51) | 1.33 (1.23;1.43) | 1.25 (1.11;1.41) | 1.35 (1.17;1.55) | 1.20 (1.07;1.34) | 1.35 (1.16;1.58) |
| **Temperature** | 1.37 (1.26;1.50) | 1.29 (1.17;1.41) | 1.14 (0.93;1.39) | 1.33 (1.23;1.44) | 1.38 (1.16;1.64) | 1.20 (1.10;1.32) |
| **Temperature range** | 1.19 (1.07;1.33) | 1.47 (1.33;1.62) | 1.21 (1.03;1.43) | 1.33 (1.21;1.47) | 1.35 (1.14;1.60) | 1.20 (1.07;1.34) |
| **Linguistic Integration** | 1.43 (1.27;1.62) | 1.29 (1.20;1.39) | 1.31 (1.16;1.48) | 1.28 (1.15;1.42) | 1.26 (1.12;1.40) | 1.24 (1.10;1.41) |
| **Foreign population** | 1.43 (1.33;1.55) | 1.20 (1.09;1.32) | 1.21 (1.05 ;1.41) | 1.35 (1.20;1.52) | 1.25 (1.09;1.43) | 1.25 (1.10;1.44) |

**Table S5.** Heat-related mortality risks (relative risk (RR) and 95% confidence interval (CI)) for high (95^th^ percentile) and low (5^th^ percentile) levels of each vulnerability factor by urban, peri-urban and rural area in Switzerland. Heat-related mortality risk corresponds to the RR at the 99^th^ percentile versus the temperature of minimum mortality.

|  | **Urban** | | **Peri-urban** | | **Rural** | |
| --- | --- | --- | --- | --- | --- | --- |
| **Variable** | **RR low (95%CI)** | **RR high (95%CI)** | **RR low (95%CI)** | **RR high (95%CI)** | **RR low (95%CI)** | **RR high (95%CI)** |
| **Ageing** | 1.18 (1.05;1.32) | 1.16 (1.05;1.27) | 1.00 (0.87;1.14) | 1.10 (1.01;1.21) | 1.13 (0.98;1.31) | 0.92 (0.76;1.12) |
| **Density** | 1.18 (1.10;1.28) | 1.14 (1.06;1.22) | 1.06 (0.93;1.21) | 1.07 (0.98;1.18) | 0.97 (0.83;1.14) | 1.11 (0.97;1.28) |
| **% of new houses** | 1.15 (1.01;1.27) | 1.17 (1.05;1.36) | 1.10 (0.99;1.23) | 1.00 (0.85;1.17) | 1.01 (0.86;1.18) | 1.08 (0.90;1.31) |
| **Social index** | 1.10 (1.02;1.30) | 1.22 (1.03;1.33) | 1.12 (1.01;1.25) | 0.99 (0.85;1.15) | 0.97 (0.80;1.16) | 1.12 (0.94;1.34) |
| **PM2.5** | 1.09 (0.98;1.23) | 1.21 (1.10;1.36) | 1.07 (0.95;1.22) | 1.06 (0.96;1.17) | 0.99 (0.82;1.20) | 1.08 (0.93;1.26) |
| **Time to health care** | 1.14 (1.05 ;1.23) | 1.22 (1.04;1.42) | 1.07 (1.00;1.14) | 1.03 (0.84;1.26) | 1.08 (0.96;1.21) | 0.93 (0.71;1.23) |
| **Impervious surfaces** | 1.20 (1.11;1.31) | 1.11 (1.02;1.21) | 1.03 (0.91;1.17) | 1.10 (0.99;1.21) | 0.98 (0.83;1.1.16) | 1.11 (0.94;1.30) |
| **EVI** | 1.08 (0.99;1.19) | 1.22 (1.12;1.34) | 1.06 0.91;1.24) | 1.07 (0.97;1.18) | 1.05 (0.89;1.25) | 1.03 (0.88;1.21) |
| **Wet bodies** | 1.19 (1.07;1.31) | 1.12 (0.99;1.28) | 1.06 0.98;1.15) | 1.07 (0.89;1.29) | 1.03 (0.92;1.16) | 1.06 (0.89;1.26) |
| **Loneliness** | 1.22 (1.09;1.36) | 1.13 (1.04;1.23) | 1.00 (0.90;1.13) | 1.15 (0.99;1.32) | 1.13 (0.97;1.31) | 0.95 (0.78;1.15) |
| **Temperature** | 1.10 (0.99;1.21) | 1.23 (1.11;1.36) | 1.07 (0.88;1.31) | 1.06 (0.99;1.15) | 0.91 (0.72;1.16) | 1.11 (0.97;1.25) |
| **Temperature range** | 1.07 (0.97;1.18) | 1.24 (1.13;1.37) | 1.11 (0.94;1.31) | 1.05 (0.95;1.15) | 0.93 (0.75;1.15) | 1.13 (0.97;1.31) |
| **Linguistic Integration** | 1.18 (1.05;1.33) | 1.14 (1.05;1.24) | 1.05(0.94;1.18) | 1.08 (0.97;1.20) | 1.01 (0.87;1.16) | 1.09 (0.92;1.30) |
| **Foreign population** | 1.16 (1.06;1.27) | 1.15 (1.02;1.30) | 1.00 (0.88;1.15) | 1.12 (0.99;1.25) | 0.99 (0.83;1.18) | 1.10 (0.92;1.31) |

|  | **Urban** | **Peri-urban** | **Rural** |
| --- | --- | --- | --- |
| **Ageing** | 0.88 | 0.62 | 0.16 |
| **Density** | 0.01 | 0.12 | 0.53 |
| **% of new houses** | 0.96 | 0.32 | 0.99 |
| **Social index** | 0.16 | 0.44 | 0.37 |
| **PM2.5** | 0.52 | 0.44 | 0.82 |
| **Time to health care** | 0.07 | 0.76 | 0.39 |
| **Impervious surfaces** | 0.002 | 0.10 | 0.70 |
| **EVI** | <0.001 | 0.51 | 0.96 |
| **Wet bodies** | 0.42 | 0.84 | 0.71 |
| **Loneliness** | 0.63 | 0.28 | 0.09 |
| **Temperature** | 0.09 | 0.43 | 0.34 |
| **Temperature range** | 0.07 | 0.67 | 0.43 |
| **Linguistic Integration** | 0.12 | 1.0 | 0.91 |
| **Foreign population** | 0.001 | 0.47 | 0.84 |

**Table S6.** Results of the Wald-test based of the exposure response curve between high and low exposure by vulnerability factor for urban, peri-urban and rural regions.

***Figure S1:* Population distribution for Switzerland at a 1x1 km resolution**


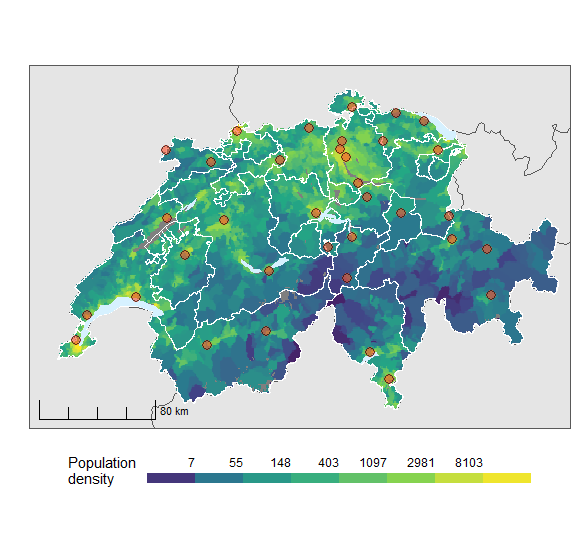


***Figure S2:* Elevation for Switzerland at a 1x1 km resolution**


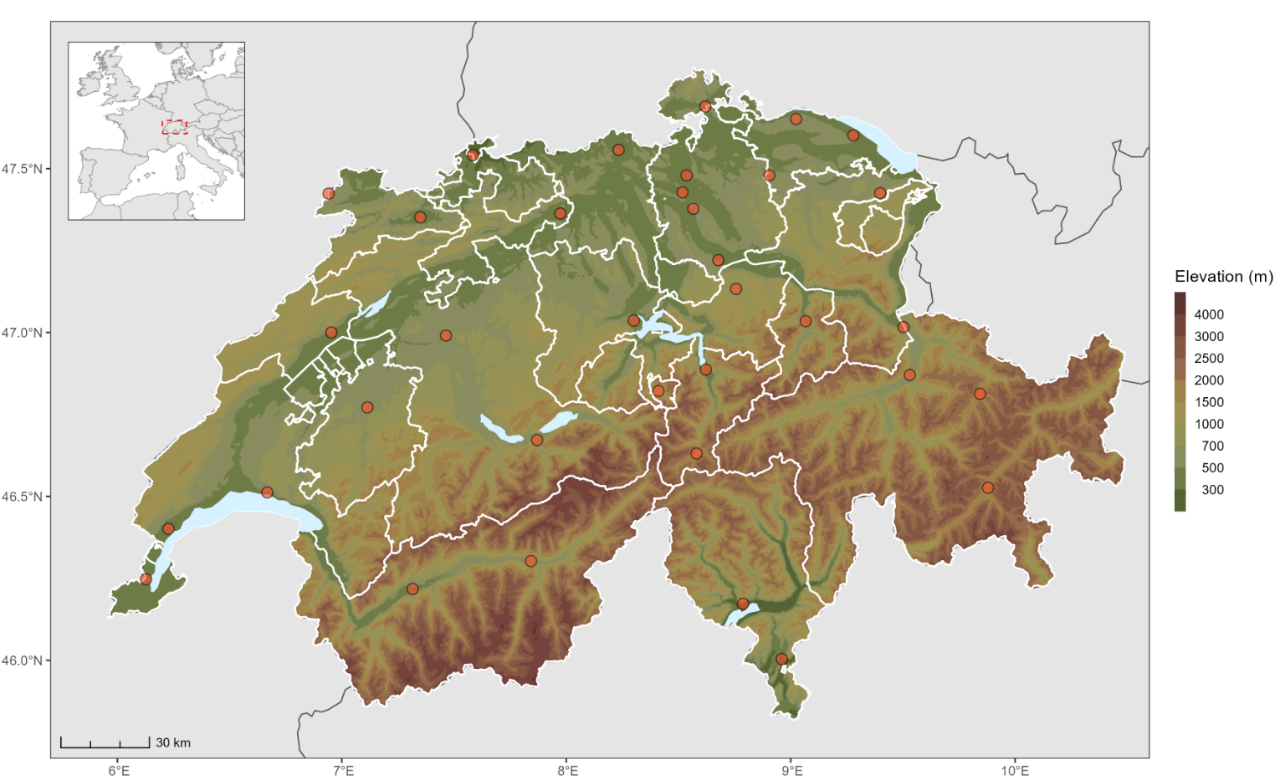


**Figure S3.** Spatial distribution of 42 collected vulnerability factors at municipality-level resolution in Switzerland

**
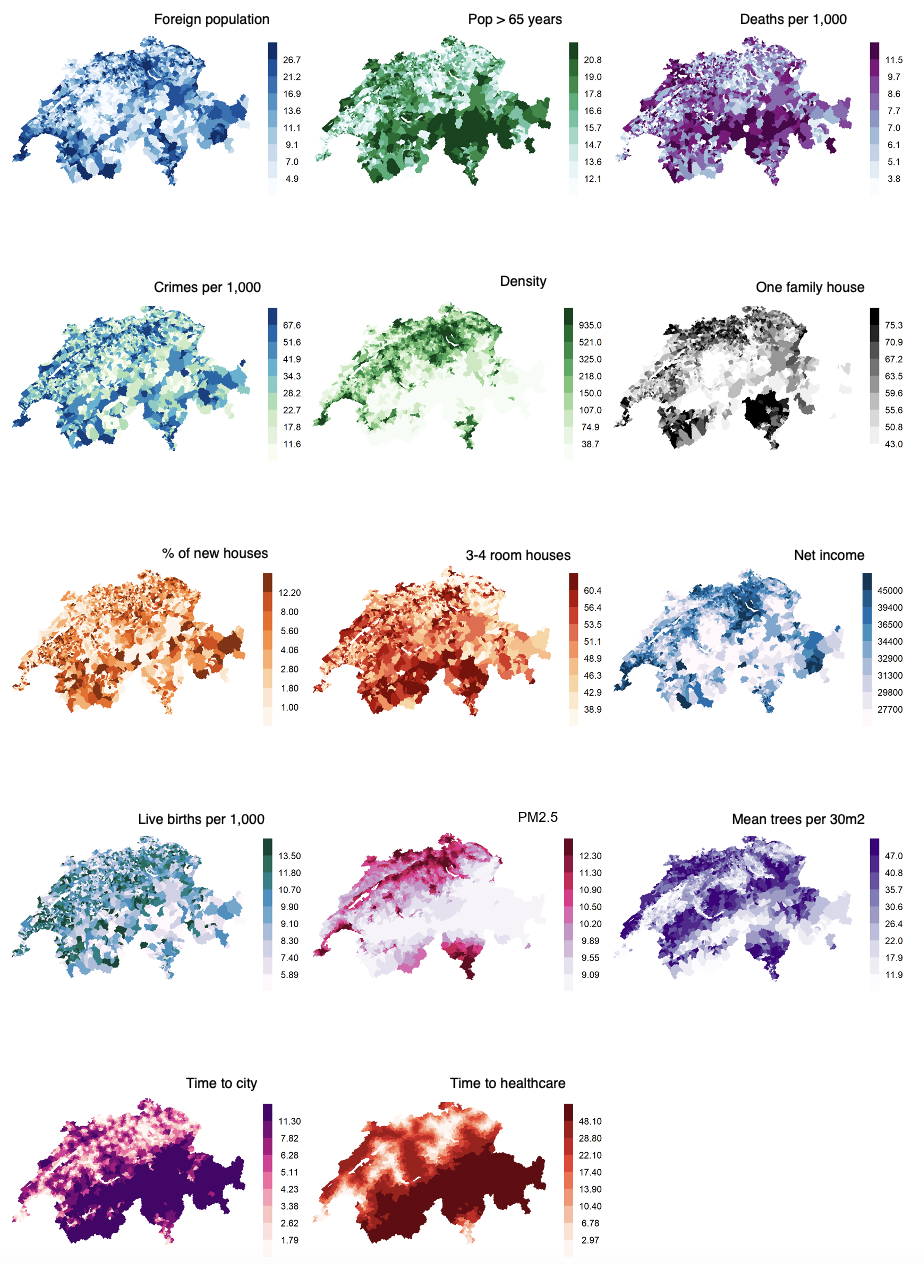
**

**
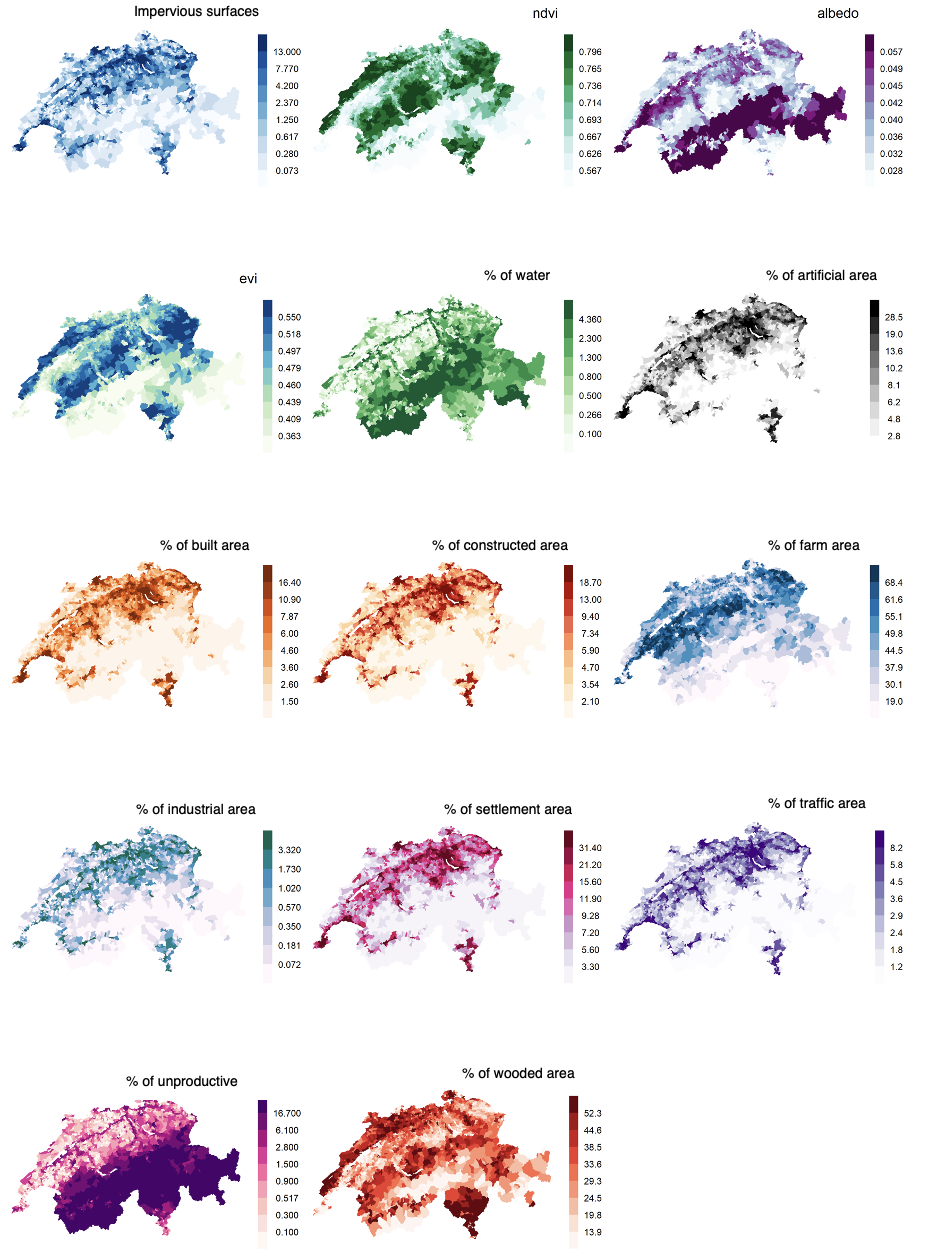
**

Temperature range

Temperature

Social index

Ageing index

Linguistic intergr.

Individualization index

3^rd^ sector employment

mean household size

4-person household

5-person household

**
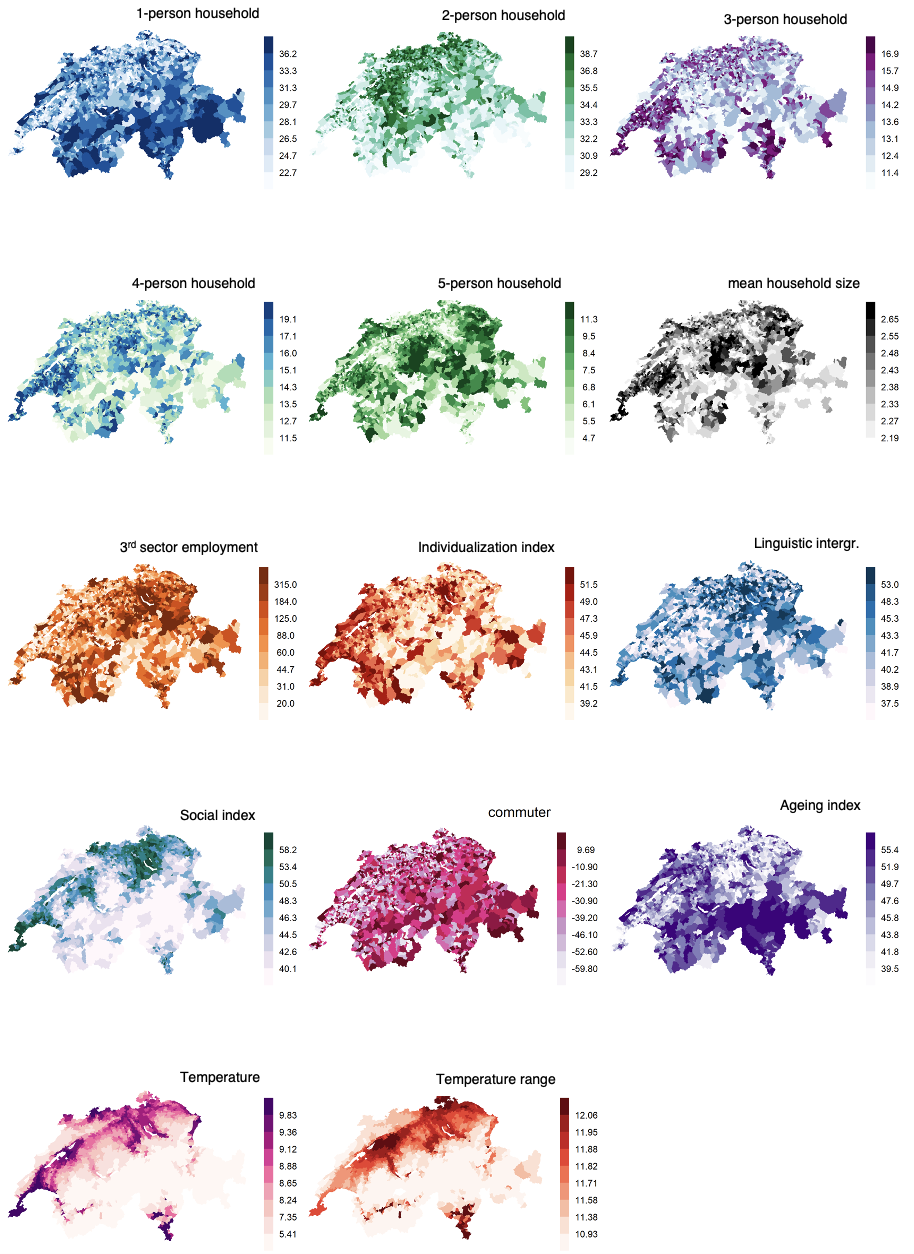
**

**Figure S4.** Correlation matrix for all 42 collected vulnerability factors at district level

**
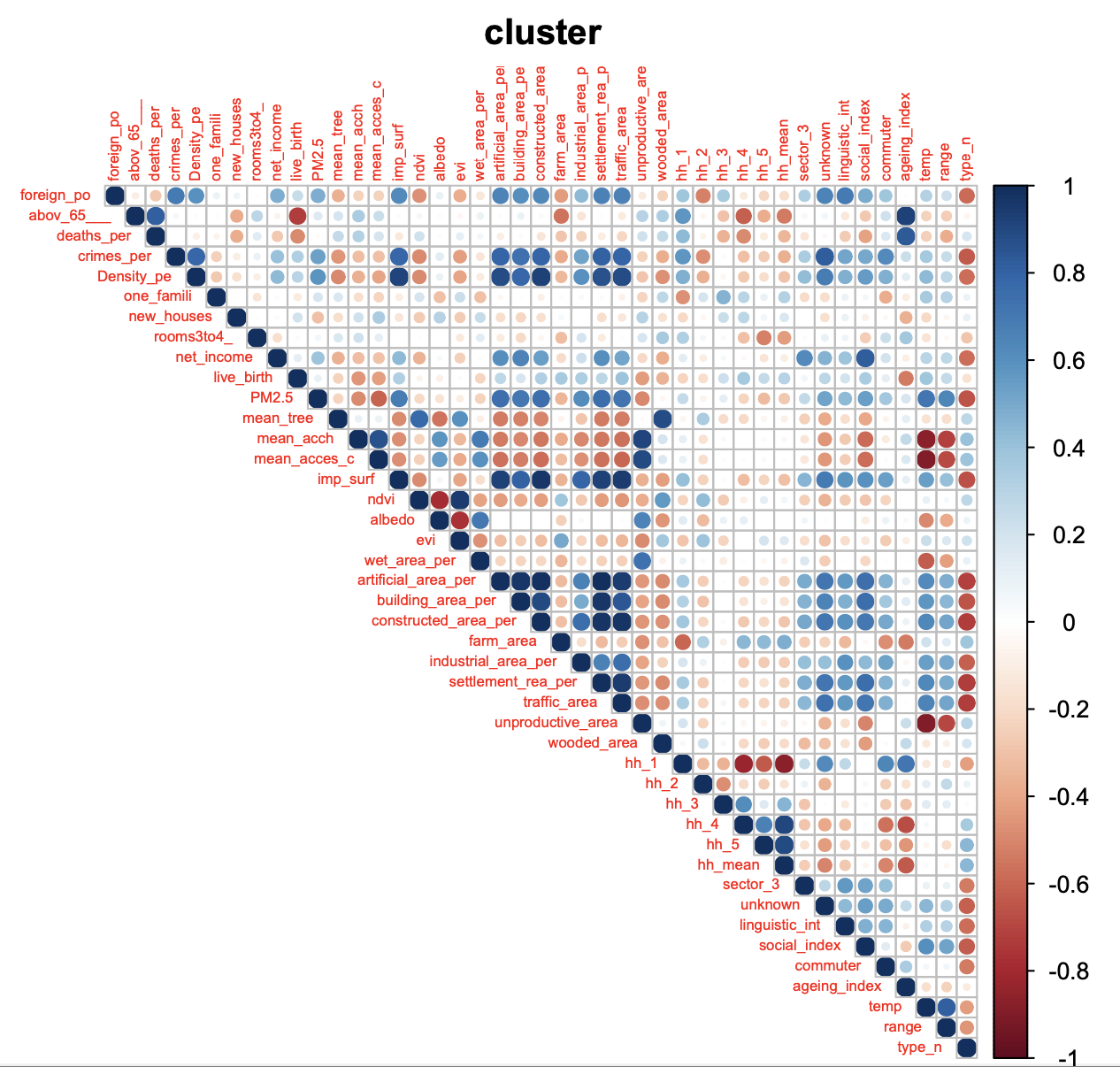
**

**Figure S5. Coordinates of selected variables**


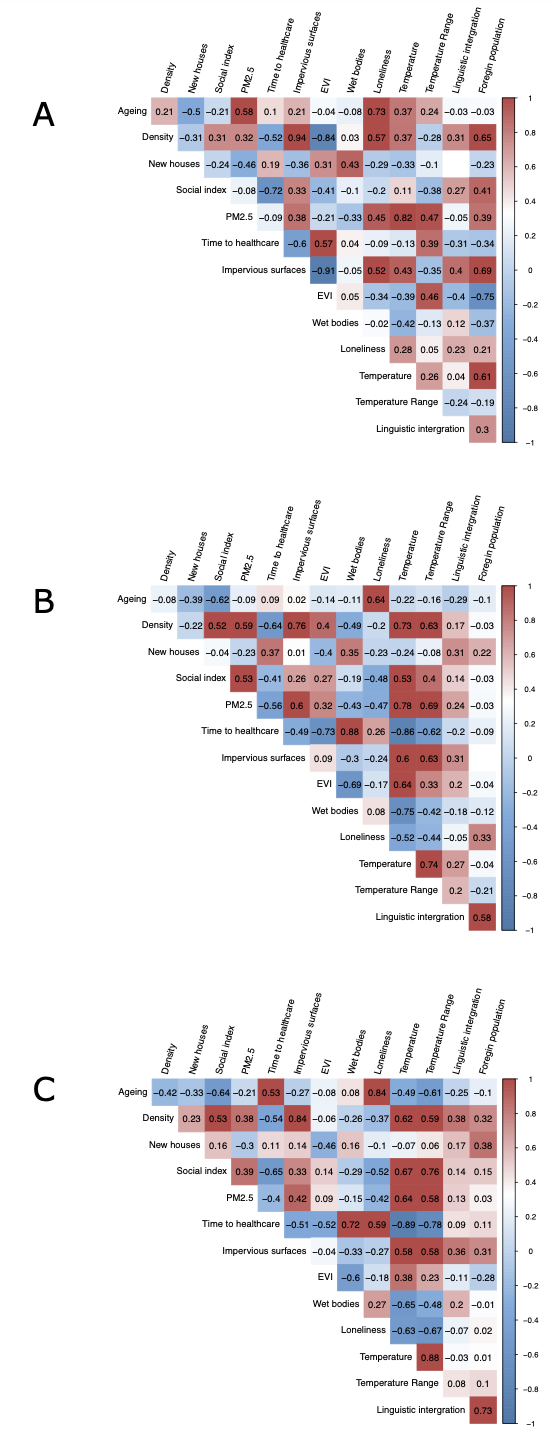
Of the 42 variables selected for the overall dataset, many of them showed a large degree of multi-collinearity (A). As a result, we picked 9 variables and picked those that were considered representative of different features and uncorrelated (B). Consequently we reduced dimensionality by conducting a principal component analysis (PCA) over these selected vulnerability factors and created two principal components. These two components were used to account for within-area typology specific confounders when predicting the urban, peri-urban and rural temperature-mortality association (as discussed in section 2.4). Then, as described in section 2.5, we predicted in univariate meta-analytical models the pooled exposure-response curves at the 5^th^ percentile (corresponding to a “low” value) and 95^th^ percentile (a “high” value) value for each of the 9 variables in each type of area. The coordinates can help us better understand which variables are correlated (A,B). The coordinates of all variables are shown in A, and indicated with a cluster group to explore the data. In B, the 9 selected variables are represented and indicated with a colour, depending on the contribution. A higher contribution, means more of the variability between clusters this variable can explain (B).


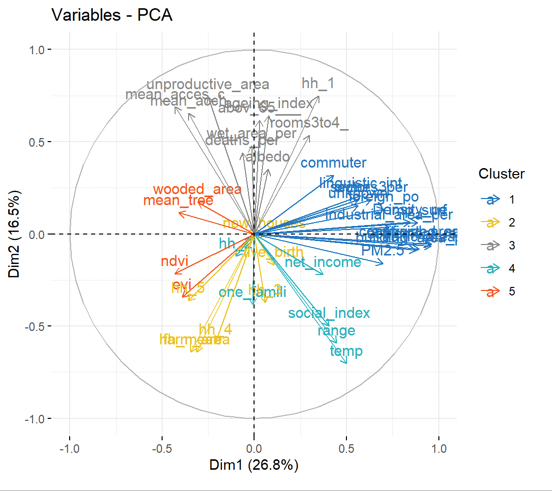

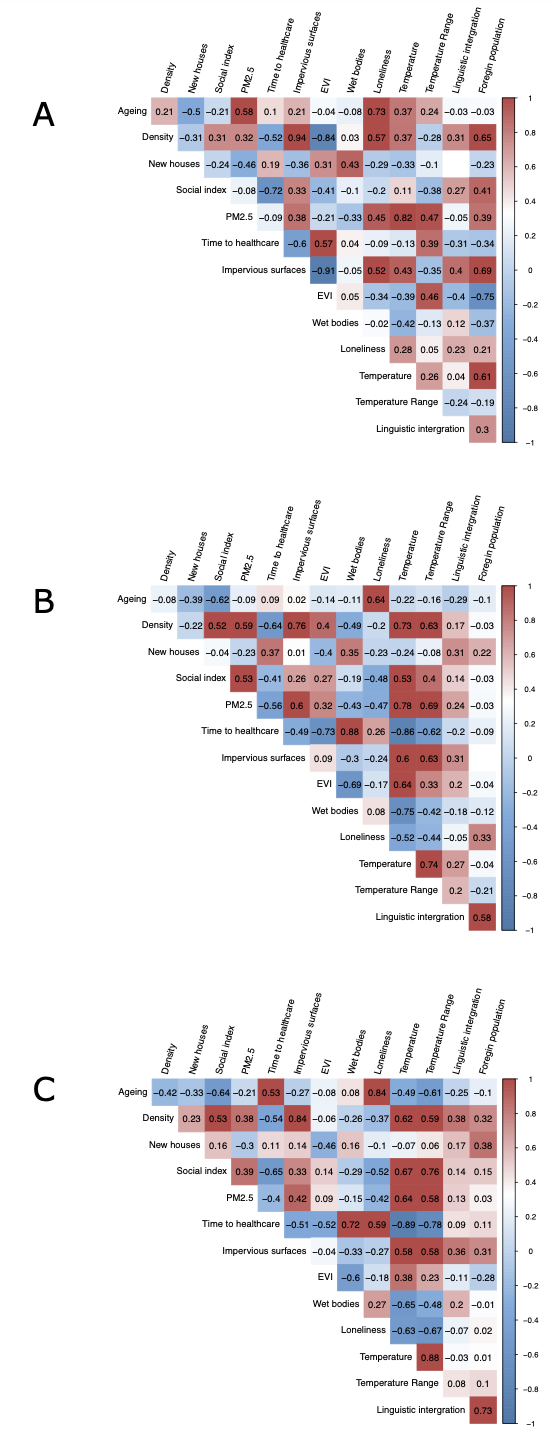
**Figure S6**. **Correlation between the selected vulnerability factors in urban (A), peri-urban (B) and rural (C) clusters.** Here we used the cluster specific data for created and computed the correlation between each vulnerability factor stratified by urban, peri-urban and rural regions.

**
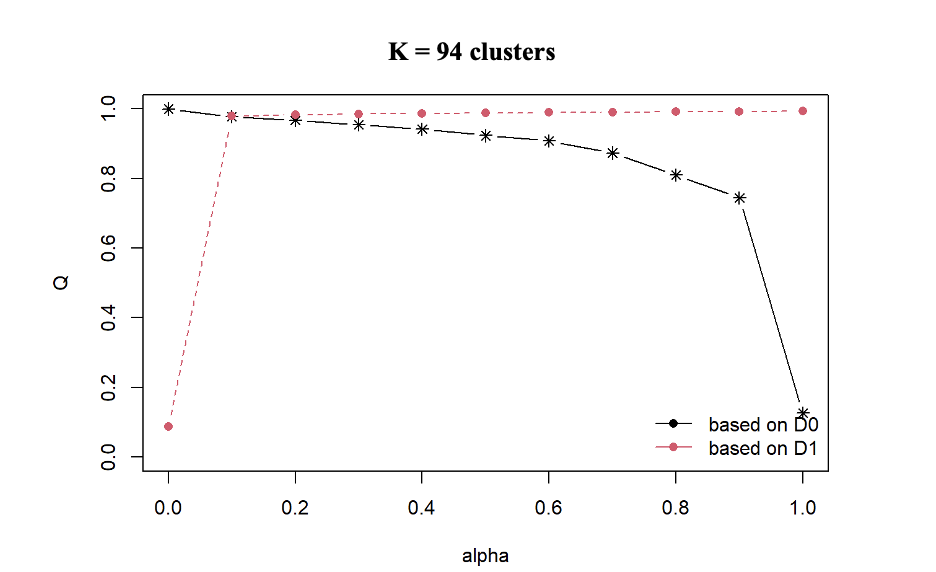
**

**Figure S7*:* Mixing parameter alpha between Matrix D_0_ and D_1_ for different values in 94 clusters.**

Matrix D_0_ = [d_0_,_ij_] is constructed based on the Manhattan distance matrix of 2,054 municipalities based on six selected spatial and demographic variables (i.e. temperature, temperature range, ageing index, social index, density and impervious surfaces). Matrix D_1_ = [d_1_,_ij_], which represented the spatial constraint space which was based on the geographical distance between the coordinates of the corresponding municipalities centroids. The inclusion of alpha assigns the relative importance of D_0_ and D_1_. When α = 0 (resp. α = 1), the hierarchical clustering is only based on matrix D_0_ (resp. = D_1_). We set α = 0.7, as this value increased spatial homogeneity without substantially reducing the quality of the solution of matrix D_0_, or the spatial-demographic variables.

**Figure S8.** Exposure-response curves for high (95^th^ percentile) and low (5^th^ percentile) levels of vulnerability factors by urban districts in Switzerland.

**Urban**

**Figure S9.** Exposure-response curves for high (95^th^ percentile) and low (5^th^ percentile) levels of vulnerability factors by peri-urban districts in Switzerland.

**Peri-urban**

**Figure S10.** Exposure-response curves for high (95^th^ percentile) and low (5^th^ percentile) levels of vulnerability factors by rural districts in Switzerland.

**Rural**

**
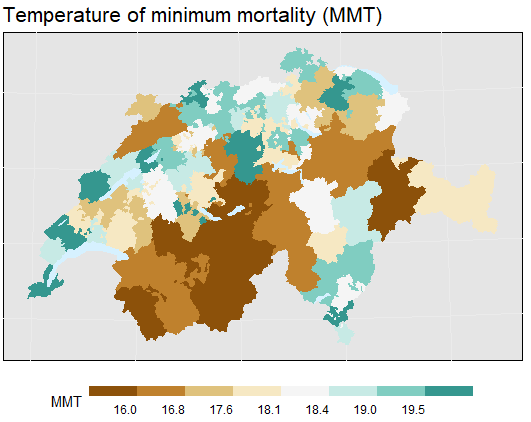
**

**References**

Center for International Earth Science Information Network – CIESIN – Columbia University. (2018). Gridded population of the world, version 4 (GPWv4): Population count adjusted to match 2010 revision of UN WPP country totals, Revision 11. Palisades, NY: NASA Socio- economic Data and Applications Center (SEDAC). https://doi.org/10.7927/H4PN93PB

Chavent, M., Kuentz-Simonet, V., Labenne, A., & Saracco, J. (2018b). ClustGeo: An R package for hierarchical clustering with spatial constraints. *Computational Statistics*, *33*(4), 1799–1822. https://doi.org/10.1007/s00180-018-0791-1

Federal Office of Topography Swisstopo (2021). SwissALTI3D. Available from: https://www.swisstopo.admin.ch/en/geodata/height/alti3d.html

Hollister, J., Shah, T., Robitaille, A. L., Beck, M., Johnson, M. (2021) Elevatr: Access Elevation Data from Various APIs. R Package. Version 0.4.1 https://cran.r-project.org/web/packages/elevatr/elevatr.pdf

van Donkelaar, A., Martin, R. V., Brauer, M., Hsu, N. C., Kahn, R. A., Levy, R. C., Lyapustin, A., Sayer, A. M., & Winker, D. M. (2016). Global Estimates of Fine Particulate Matter using a Combined Geophysical-Statistical Method with Information from Satellites, Models, and Monitors. *Environmental Science & Technology*, *50*(7), 3762–3772. https://doi.org/10.1021/acs.est.5b05833

Weiss, D. J., Nelson, A., Vargas-Ruiz, C. A., Gligorić, K., Bavadekar, S., Gabrilovich, E., Bertozzi-Villa, A., Rozier, J., Gibson, H. S., Shekel, T., Kamath, C., Lieber, A., Schulman, K., Shao, Y., Qarkaxhija, V., Nandi, A. K., Keddie, S. H., Rumisha, S., Amratia, P., … Gething, P. W. (2020). Global maps of travel time to healthcare facilities. *Nature Medicine*, *26*(12), 1835–1838. https://doi.org/10.1038/s41591-020-1059-1
